# Decamethylcyclopentasiloxane (D5) Oxidation: Product Chemistry, Influence of RO_2_ Fate, and Secondary Aerosol Production

**DOI:** 10.1021/acsestair.6c00037

**Published:** 2026-07-10

**Authors:** Saeideh Mohammadi, Jeewani N. Meepage, Christopher E. Brunet, Rachel F. Marek, Keri C. Hornbuckle, Eleanor C. Browne, Elizabeth A. Stone, Charles O. Stanier

**Affiliations:** † Department of Chemical and Biochemical Engineering, 4083 University of Iowa, Iowa City, Iowa 52242, United States; ‡ IIHR-Hydroscience and Engineering, 4083 University of Iowa, Iowa City, Iowa 52242, United States; § Department of Chemistry, 4083 University of Iowa, Iowa City, Iowa 52242, United States; ∥ Department of Civil and Environmental Engineering, 4083 University of Iowa, Iowa City, Iowa 52242, United States; ⊥ Department of Chemistry and Cooperative Institute for Research in Environmental Sciences, 1877 University of Colorado, Boulder, Colorado 80309, United States

**Keywords:** volatile methylsiloxanes (VMS), decamethylcyclopentasiloxane (D_5_), oxidation flow reactor (OFR), siloxanols, RO_2_ chemistry, secondary organic aerosol (SOA), multigenerational oxidation, volatility basis set (VBS) modeling

## Abstract

Volatile methylsiloxanes (VMS), particularly decamethylcyclopentasiloxane (D_5_), are recognized anthropogenic precursors of secondary organic aerosol (SOA), yet their formation pathways, product distributions, and yields under atmospherically relevant conditions remain poorly constrained. This study examines D_5_-derived SOA across a wide range of OH exposures (OH_exp_) and peroxy radical (RO_2_) fates (RO_2_+HO_2_ and RO_2_+OH) in an oxidation flow reactor, with comparison to ambient and chamber samples. The siloxanol (1-hydroxynonamethylcyclopentasiloxane, D_4_TOH) is consistently observed. Under low OH_exp_ and HO_2_-dominated regimes, samples contained multiple early generation siloxanols accompanied by dimers and high molecular weight products, contrary to expectations that such species require extensive oxidation. As OH_exp_ and RO_2_ + OH reactivity increase, total SOA mass rises, and the fraction of unresolved material increases. The observations were compared to a volatility basis set (VBS) multigenerational oxidation kinetic box model with explicit siloxanols. The model’s strongly monotonic progression in volatility, aerosol yield, and degree of OH substitution in the polysiloxanol series diverged from experimental data, aside from the expected increase in yield with OH_exp_. At high OH_exp_, the product distribution and yield appear to be insensitive to RO_2_+HO_2_ vs RO_2_+OH fate.

## Graphical abstract

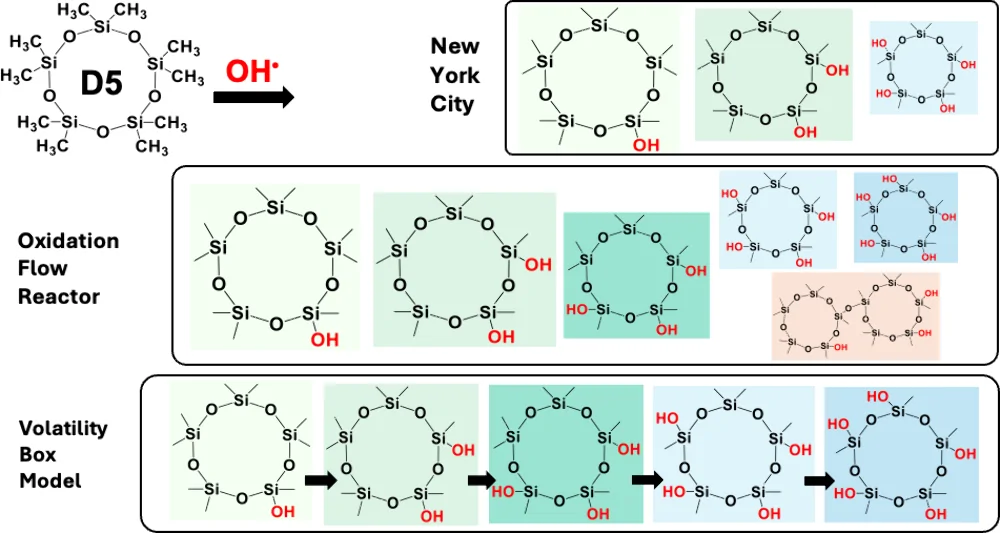

## 1 Introduction

With vehicular volatile organic compound (VOC) emissions declining markedly in recent decades,^1,2^ volatile chemical products (VCPs) have emerged as a major urban VOC source and are now recognized as precursors of secondary organic aerosol (SOA).^3–5^ Among VCPs, cyclic volatile methyl siloxanes (cVMS), especially decamethylcyclopentasiloxane (D_5_; C_10_H_30_O_5_Si_5_), are notable for their widespread use in industrial and personal care products (PCP),^6^ high emission rates, and unique atmospheric behavior.^4,7,8^ D_5_ volatilizes readily and undergoes limited wet and dry deposition removal – leading to atmospheric accumulation,^9^ prompting regulatory scrutiny, and motivating the need to understand its gas- and particle phase atmospheric transformations.^10–12^

The dominant atmospheric sink for D_5_ is gas-phase oxidation by hydroxyl (OH) radicals, with a rate constant of ∼2.0 × 10^–12^ cm^3^ molecule^–1^ s^–1^, corresponding to a 4–5 day atmospheric lifetime under typical OH levels.^13,14^ Chlorine (Cl) radical oxidation supplements OH, contributing ∼18% of the global sink,^15^ while reactions with ozone (O_3_) and nitrate radicals (NO_3_) are negligible.^14^ D_5_’s long atmospheric lifetime enables long-range transport – supported by remote detections in the Arctic^16^ and Tibetan Plateau^17^ – making its gas-phase chemistry central to its removal and SOA formation.^18–20^

Both field and laboratory studies support the formation of D_5_-derived SOA. Ambient measurements across multiple locations confirm the presence of oxidized D_5_ products in fine particulate matter (PM_2.5_).^21–23^ Laboratory studies have investigated SOA formation from D_5_ in both environmental chambers^24–30^ and oxidation flow reactors (OFRs),^31–34^ with siloxanol (C_9_H_28_O_6_Si_5_, 1-hydroxynonamethylcyclopentasiloxane, or D_4_TOH) reported as the dominant oxidation product.^23,24,28–30^ Siloxanol is formed through the substitution of one methyl group with a hydroxyl group on the D_5_ ring. Ambient PM_2.5_ detections of siloxanol, coupled with its unique chemical signature and anthropogenic origin, have led researchers to propose siloxanol as a tracer of PCPs.^21,26,35^ The product distribution shifts with increasing OH exposure (OH_exp_), with disiloxanols, additional functionalized products, and ring-opened and oligomeric species detected, consistent with multigenerational aging.^30,34^

Despite substantial progress in characterizing D_5_ oxidation chemistry, uncertainties remain regarding how environmental conditions control SOA yields and product evolution, particularly under the accelerated oxidative environments of OFRs. Although OFRs are widely employed to simulate atmospheric aging, SOA formation in these systems is influenced by several interdependent parameters beyond precursor reactivity including OH_exp_, relative humidity (RH), residence time (τ), and surface losses to reactor walls. These factors influence gas-particle partitioning, oxidation pathways, and particle phase aging.^36–38^ Chamber and OFR studies consistently report increasing SOA yields from D_5_ (from <0.01 to ∼1.5) with increasing OH_exp_ across a wide range of conditions,^25,28–34^ indicating that functionalization dominates and fragmentation plays a minor role. These SOA yields vary with experimental parameters such as RH, NO_*x*_ levels, seed aerosol, and precursor loading, which influence the product distribution and gas-to-particle partitioning.

Importantly, the oxidative environment within OFRs differs significantly from that of ambient atmospheric conditions. Short residence times and high radical concentrations, particularly OH, can alter the fate of organic peroxy radicals (RO_2_). Whereas RO_2_ + HO_2_ reactions are typical under low-NO_*x*_ ambient conditions,^39,40^ RO_2_ reactions with OH or other RO_2_ radicals can be favored in OFRs.^41,42^ These alternative pathways may influence product distributions and SOA yields. While the RO_2_ fate has been shown to influence VOC oxidation chemistry more broadly, its specific role in siloxane-derived SOA formation remains poorly constrained. Although siloxanols are consistently observed as early oxidation products, it remains unclear how further functionalization, condensation, or fragmentation governs SOA formation under different oxidative regimes. Existing modeling frameworks primarily emphasize gas-phase pathways, further limiting the mechanistic understanding of siloxane aging.

To address these uncertainties, this study investigates how OH_exp_ and RO_2_ fate govern SOA formation and oxidation product distributions from D_5_ oxidation in an OFR. Specifically, we aim to (i) quantify SOA formation across a controlled range of OH_exp_ and RO_2_ fates; (ii) identify and classify key oxidation products and their volatilities using ultraperformance liquid chromatography–tandem mass spectrometry (UPLC-MS); and (iii) assess the extent to which OH_exp_ and RO_2_ fate influence the mass and chemical composition of D_5_-derived SOA. The results are intended to clarify D_5_ oxidation mechanisms and improve the representation of VCP-derived SOA yield and product distribution in chemical transport models.

## 2 Methods and Experiments

### 2.1 Oxidation Flow Reactor (OFR) Experiments

Experiments were conducted using an Aerodyne Potential Aerosol Mass (PAM) Oxidation Flow Reactor (OFR185 mode), as described in prior studies.^37,43,44^ The experimental setup is illustrated in Figure S1. The OFR was operated under low-NO_*x*_ conditions with a centerline sample flow of 3.5 L min^–1^, complemented by a ring flow of 1 L min^–1^, yielding a total flow rate of ∼4.5 L min^–1^. The ring flow was discarded to minimize sampling of air parcels influenced by wall effects. The centerline was sampled for gas and aerosol analyses.^37,43^ Flow dynamics were optimized to achieve minimal recirculation; the actual mean residence time was determined to be ∼156 s based on residence time distribution (RTD) testing with pulses of monodisperse 200 nm ammonium sulfate. UV lamp settings ranged from 0.4 to 8.0 V, modulating photon flux and OH generation rates. Although the OFR is equipped with RH sensors for real-time monitoring, the relative humidity of the incoming airflow was independently verified upstream using a Rotronic humidity probe. In experiments using carbon monoxide (CO) as an external source of OH reactivity (OHR_ext_),^41,45^ a controlled flow rate of 1,000 ppm of CO (ProSpec, Linde) was diluted in dry air using mass flowmeters (Alicat Scientific) to achieve the desired concentration at the OFR inlet. Table S1 lists key details from each experiment. Seven experiments are listed in Table 1 with chemical composition analysis of aerosol and/or gas phase products for the core of the analysis and discussion.

**Table 1:** OFR Experimental Conditions for Seven Selected Tests in the Range of OH_exp_ and RO_2_ Loss Pathways

**Exp**	**Sample**ID	**OH**_**exp**_ **× 10** ^**11**^ **(cm** ^**‑3**^ **·s)**	**Eq Aging** ^b^(days)	**HO**_**2**_ **/**OH	**RO**_**2**_ **+ OH Fraction**	**RO**_**2**_ **+ HO**_**2**_ **Fraction**	**ΔD**_**5**_ **(μg m** ^**‑3**^ **)**	**SOA Mass** **(μg m** ^**‑3**^ **)**	**SOA Yield**	**Model SOA Yield**	**Samples**
**1**	16-CO	4.59	4.4	97	0.06	0.94	945	50 ± 2	0.05	0.19	QFF/PUF^a^
**2**	15	5.17	5.0	11	0.34	0.65	938	42 ± 2	0.04	0.27	QFF/PUF^a^
**3**	17-CO	10.3	9.9	55	0.09	0.91	2182	101 ± 2	0.05	0.64	QFF/QFF/PUF
**4**	14	18.9	18	9.2	0.56	0.44	1104	78 ± 3	0.07	0.85	QFF/PUF
**5**	13	94.2	90.9	11	0.53	0.47	1411	215 ± 5	0.15	1.00	QFF/PUF
**15**	E10-CO	4.7	5	122	0.05	0.95	1427	19 ± 1	0.01	0.98	N/A
**16**	E10	32.1	31	10	0.47	0.53	1457	140 ± 3	0.10	1.00	N/A

a OC analysis was performed on these experiments.

b Eq Aging = Equivalent Aging

To introduce D_5_ at desired mixing ratios into the OFR, a solution of D_5_ diluted in carbon tetrachloride was injected into a flow of RH-controlled air using a syringe pump. Gas-phase D_5_ samples were collected upstream and downstream of the OFR onto ISOLUTE ENV+ 10 mg mL^–1^ cartridges at controlled flow rates and were analyzed using an Agilent 7250 GC/Q-TOF system operating in MS/MS mode. The mass of reacted D_5_ was calculated from the difference between upstream and downstream concentrations and used to compute SOA mass yield for each experiment. The Supporting Information (Section S2) contains additional details on standard preparation, extraction, instrumental analysis, and quantification. To detect low-concentration impurities, the D_5_ stock solution and selected gas-phase samples were analyzed using a multiple-reaction-monitoring method on an Agilent 7000D GC-MS/MS Triple Quad as described previously,^46^ with details in Section S7.

### 2.2 Particle Measurements and SOA Analysis

Aerosol size distributions and number concentrations were measured using a TSI 3936L85 scanning mobility particle sizer (SMPS), consisting of a TSI 3080 electrostatic classifier, TSI 3081 long-column differential mobility analyzer, TSI 3025A condensation particle counter, and a TSI 3077 Kr-85 neutralizer (2 mCi). The SMPS operated with a sheath flow rate of 3 L min^–1^ and an aerosol flow rate of 0.3 L min^–1^ and covered a mobility diameter range of 15.1 to 661.2 nm. Size distributions were used to calculate particle volume concentrations, assuming a spherical morphology. SOA mass concentrations were derived by applying a density of 1.2 g cm^–3^, determined gravimetrically from Teflon filter samples collected downstream of the OFR, as described in Section S3.

Wall losses in PAM OFR have been reported to be generally lower than in static environmental chambers due to their continuous flow and short residence times. However, it remains a measurable factor, especially for ultrafine particles and semivolatile gases.^38^ Moreover, mechanisms such as diffusional deposition, gravitational settling, and inertial losses in bends can contribute to size-dependent transmission loss. The sampling system in this study was designed to minimize such effects through short tubing lengths, smooth bends, and moderate flow rates. Estimated transmission efficiencies based on standard transport models^47^ indicated >95% transmission for particles larger than 50 nm and >99% for accumulation-mode particles, with only minor losses in the nucleation mode. Given the low contribution of sub-50 nm particles to total mass, the influence of sampling losses on integrated mass concentrations was deemed negligible. Therefore, SMPS distributions, without loss correction, were used for quantifying SOA formation and particle growth. SOA yields were calculated as the ratio of particle phase mass to the mass of reacted D_5_ (ΔD_5_). The OH_exp_ was controlled in the OFR experiments by adjusting UV lamp power, RH, and OHR_ext_. OH_exp_ was estimated using the kinetic model as the time integrated OH concentration over the residence time of the flow reactor.

### 2.3 Oxidation Product Chemical Characterization

#### 2.3.1 Sample Collection, Preparation, and Analysis

Offline chemical characterization followed the method described by Meepage et al. (2024, hereafter M24).^21^ Sample collection was configured for the OFR system and is briefly described here. Particle phase and semivolatile oxidation products were collected downstream of the OFR using a sampling setup as shown in Figure S5. Quartz fiber filters (QFF, 47 mm, PALL Life Sciences) captured particulate matter, while polyurethane foam filters (PUF; URG-2000–30–52PC) were used to trap semivolatile organic compounds (SVOC). A total sampling flow rate of 3.5 ± 0.2 L min^–1^ was maintained, and all flow paths used 1/4” OD PTFE tubing with stainless steel connectors to minimize wall/tubing artifacts. All QFFs were prebaked at 550 °C for 18 h, and PUF filters were precleaned with ultrapure water, acetone, and acetonitrile by sonication and repeated compression. Each sampling train was leak-checked and flow-balanced prior to experiments to ensure reproducibility. To estimate potential positive sampling artifacts associated with QFF collection, two precleaned QFFs were placed in series during Experiment 17-CO. The second QFF was placed to capture any particulate matter that broke through the first filter and/or to adsorb gases that would sorb to both front and back filters.

Analysis of oxidation products utilized a UPLC-MS equipped with a reversed-phase C18 column (ACQUITY BEH, 2.1 × 100 mm, 1.7 μm particle size, Waters) and high-resolution Q-Exactive Quadrupole Orbitrap mass spectrometer operated in negative electrospray ionization mode (ESI(−)). Molecular formulas were assigned to detected species by matching their *m*/*z* values (within ±5 ppm of theoretical values) and isotope distribution patterns. Targeted analysis focused on 135 compounds, shown in Figure S5, identified in prior studies, with additional criteria applied to account for potential modifications, such as hydroxyl or silylmethanol substitutions. Semiquantitation was performed using TraceFinder v5.0 (Thermo Scientific). Because direct quantification was not feasible due to the lack of authentic standards, a semiquantitative approach was applied using tris­(tert-butoxy)­silanol as a surrogate standard (99.999%, Sigma-Aldrich). This approach assumes that all oxidation products of D_5_ exhibit equivalent ionization efficiencies to the tris­(tert-butoxy)­silanol and does not account for varying desolvation efficiency as the organic mobile phase B increases from 45 to 100%, both of which can introduce biases in absolute concentrations but are not expected to affect comparisons of relative signals across samples. More details about sample preparation and chemical analysis can be found in the SI (Section S4). To evaluate background contributions from sampling media and handling, one set of QFF and PUF field blanks was collected without airflow and analyzed using the same UPLC-MS method. Measured signals on both field blanks were either at or below method detection limits. The semiquantification analysis focused on a set of oxidized products, with structural details reported in Lewine et al. (2025, hereafter L25).^28^

Previously published ambient aerosol samples collected during the NYC-METS 2022 campaign in Manhattan, New York City were analyzed using the same UPLC-MS method described above.^23^ Seven colocated QFF and PUF samples collected between July 25 and July 29, 2022, spanning alternating 12-h daytime and nighttime periods (Table S4), were processed individually and then averaged to generate a representative ambient sample for comparison with OFR conditions and box model predictions. Gas and particle phase contributions, field blanks, quantification limits, surrogate standards, semiquantification, and nondetect sensitivity analyses are found in Welker et al. (2025, hereafter W25).^23^

### 2.4 OFR Kinetic Model

A kinetic OFR model was implemented to simulate radical and photochemistry in the OFR. Kinetic parameters for OFR radical reactions, including photolysis, were from Peng and Jimenez (2019).^42^ Reactions of CO were added, using the 1 atm IUPAC rate coefficient for the CO + OH reaction rate. The model was solved in MATLAB using ode15s, a variable step, variable order stiff differential equation solver. Model reactions and rate coefficients can be found in the SI (Table S6). The model was evaluated by comparison to a previously published benchmark.^45^

The reaction mechanism for D_5_ oxidation was adapted from the multigenerational VBS oxidation model used in Kang et al. (2023, hereafter K23). The initial oxidation reaction is shown in [Disp-formula eqR1]

$$D5+\mathrm{OH}\to \alpha_{1}\left[\delta {\mathrm{SILOX}}_{1}+\left(1-\delta \right){\mathrm{SV}}_{1}\right]+\alpha_{2}{\mathrm{SV}}_{2}+\alpha_{3}{\mathrm{SV}}_{3}+\alpha_{4}{\mathrm{SV}}_{4}+\alpha_{5}{\mathrm{SV}}_{5}+\alpha_{6}{\mathrm{SV}}_{6}$$ R1

where SV_1_–SV_6_ are lumped semivolatile surrogate compounds in a volatility basis set (VBS) series of saturation concentrations. Stoichiometric coefficients are α_i_, and δ is a branching coefficient for the formation of the siloxanol (SILOX_1_) which can range from 0–1. The model reduces to the K23 model when δ = 0. In K23, the purpose of the model was to reproduce yields, irrespective of chemistry. Because we have measurements of SILOX_1_ to SILOX_5_ (the siloxanol to the pentasiloxanol) via liquid chromatography QFF/PUF measurements for the oxidation flow reactor, New York City ambient air,^23^ and chamber experiments,^28^ the K23 mechanism was adapted for siloxanol estimation. This permits model observation comparison of total mass (using the sum of the siloxanols and the semivolatile surrogate species) as well as the speciated and total siloxanols.

The multigenerational VBS bin-hopping reactions of the semivolatile surrogates are

$${\mathrm{SV}}_{n}+\mathrm{OH}\to \beta \mathrm{VP}+\left(1-\beta \right){\mathrm{SV}}_{n+1}\;\left(\mathrm{valid}\;\mathrm{for}\;n\;=\;1\;\mathrm{to}\;5\right)$$ R2

$${\mathrm{SV}}_{6}+\mathrm{OH}\to \beta \mathrm{VP}+\left(1-\beta \right){\mathrm{SV}}_{6}\;\left(\mathrm{valid}\;\mathrm{for}\;n\;=\;6\right)$$ R3

where VP is a noncondensable volatile product (i.e., a fragmentation product), and β is a fragmentation parameter ranging from 0–1. These reduce to the K23 mechanism when β = 0.

The multigenerational VBS bin-hopping reactions of the siloxanols are an extension of the successive siloxanol oxidation in L25, with a channel for production of a noncondensible species (VP) via fragmentation, and a channel for siloxanols to be oxidized by OH and produce nonsiloxanol compounds, which we track through the SV surrogates

$${\mathrm{SILOX}}_{n}+\mathrm{OH}\to \beta \mathrm{VP}+b{\mathrm{SILOX}}_{n+1}+c{\mathrm{SV}}_{n+1}\;\left(\mathrm{valid}\;\mathrm{for}\;n\;=\;1\;\mathrm{to}\;4\right)$$ R4

$${\mathrm{SILOX}}_{5}+\mathrm{OH}\to \beta \mathrm{VP}+\left(1-\beta \right){\mathrm{SV}}_{6}$$ R5

where b = δ­(1 – β) and c = (1 – δ)­(1 – β).

D_5_ reaction rates were used from both K23 and Alton and Browne (2022) with modifications as detailed below.^34,48^ Further implementation details include logarithmically spaced effective saturation concentrations (c*) of 10^4^, 10^3^, 10^2^, 10^1^, 10^0^, and 10^–1^ μg m^–3^. Oxidation products were modeled with a uniform molar mass of 373 g mol^–1^. The model did not resolve between aerosol and gas phase species until a postprocessing determination of gas-particle equilibrium partitioning using eq 1

$$f_{p,i}={\left(1+\frac{c_{i}^{*}}{c_{OA}}\right)}^{-1}$$ 1

where *f*_*p,i*_ is the fraction of species in the particle phase, *c*_i_^*^ is the saturation concentration of species *i*, and *c*_OA_ is the organic aerosol mass concentration. Saturation concentrations were derived from vapor pressures reported in Jang et al. (1997),^49^ with assignment to the nearest VBS bin. The resulting VBS assignments were c* of 10^4^, 10^3^, 10^1^, 10^0^, and 10^–1^ μg m^–3^, for siloxanols SILOX_1_ through SILOX_5_, respectively. The rate for subsequent oxidations by OH after the initial oxidation was 2.17 × 10^–12^ cm^3^ molec^–1^ s^–1^. The kinetic mechanism and implementation of the kinetic model are summarized graphically in a flowchart in Figure S11.

Lamp power and the resulting actinic flux are important variables in the kinetic box model. Details of the 185 and 254 UV lamps calibration and photon flux determination (hereafter UV_185_ and UV_254_) are provided in Section S10 of the Supporting Information. Briefly, OFR lamp irradiance was measured using a UV sensor inside the chamber under different dimming voltages (1–10 VDC). Fourth-order polynomial fits were derived for the 0–4 V and 4–10 V ranges (Figure S15), enabling accurate estimation of normalized lamp power from applied voltage (Table S10). To determine photon fluxes used in the model, irradiances were first converted to photon fluxes by dividing by the photon energy at each wavelength. The resulting fluxes were compared against published values^50,51^ and adjusted for optical attenuation in the reactor. Maximum UV_254_ and UV_185_ of 7.0 × 10^15^ and 3.2 × 10^14^ photons cm^–2^ s^–1^, corresponding to a UV_185_/UV_254_ ratio of 0.045, were established by matching modeled to measured O_3_ concentrations downstream of the OFR. These values were then used to simulate radical production, OH_exp_, and RO_2_ fates in the kinetic box model.

As a metric to assist with interpretation and comparison of model and observational results, we use a “siloxanol number” (SN), the average degree of substitution per siloxanol molecule. Two variants of SN are used in this work. We utilize the siloxanol through the pentasiloxanol to calculate SN_1–5_.

$${SN}_{1-5}=\frac{\underset{i=1\cdot \cdot \cdot 5}{\sum }i\times {SILOX}_{i}}{\underset{i=1\cdot \cdot \cdot 5}{\sum }{SILOX}_{i}}$$ 2

SN_1–5_ can vary from 1 (for 100% SILOX_1_, or siloxanol) to 5 (for 100% SILOX_5_, the pentasiloxanol). For simplicity, only simple siloxanols (molecules with only methyl and alcohol substitutions) were included in the numerator and denominator, with more complex molecules (i.e., one formate ester and one alcohol group, silyl methanols, etc.) excluded. Where model-observation is shown using SN_1–5_, sensitivity analysis was performed varying the values of any siloxanols below method detection limits to both zero and one-half of the method quantification limit of the surrogate standard. In cases where tri- through pentasiloxanols were frequently below the detection limits and therefore poorly constrained, SN_1–2_ was used instead, as it relies only on the two most abundant and quantified siloxanol species.

$${SN}_{1-2}=\frac{\underset{i=1\cdot \cdot \cdot 2}{\sum }i\times {SILOX}_{i}}{\underset{i=1\cdot \cdot \cdot 2}{\sum }{SILOX}_{i}}$$ 3

RO_2_ fate was tracked during box model integration using the procedure described below. Reactions of the VMS RO_2_ radical (VMSRO_2_) did not explicitly lead to tracked products (rather, tracked products were formed directly from the reactions with OH as shown in [Disp-formula eqR1]–[Disp-formula eqR5]). However, the fate of RO_2_ radicals was tracked as a diagnostic variable to see if it correlated with model-observation deviations. The RO_2_ fate tracking was done using [Disp-formula eqR6]–[Disp-formula eqR11]

$$D5_{z}+\mathrm{OH}\to \mathrm{OH}+{\mathrm{VMSRO}}_{2}$$ R6

where D5_*z*_ is D_5_ or any of its reaction products. In other words, we allow every reaction with OH to form an RO_2_ radical and then track the fate of those RO_2_ radicals using eqs [Disp-formula eqR7]–[Disp-formula eqR11].

$${\mathrm{VMSRO}}_{2}+\mathrm{NO}\to \mathrm{SINK}+{\mathrm{NO}}_{2}$$ R7

$${\mathrm{VMSRO}}_{2}+{\mathrm{HO}}_{2}\to \mathrm{SINK}$$ R8

$${\mathrm{VMSRO}}_{2}+{\mathrm{VMSRO}}_{2}\to \mathrm{SINK}$$ R9

$${\mathrm{VMSRO}}_{2}+\mathrm{OH}\to \mathrm{SINK}$$ R10

$${\mathrm{VMSRO}}_{2}\to \mathrm{SINK}$$ R11

Rates for these diagnostic tracking reactions were taken from Alton and Browne (2022) and L25.^28,48^ By integrating these reaction rates over the course of the OFR time scale, the relative fate of the VMS RO_2_ radical (i.e., 50% RO_2_ + OH, 45% RO_2_ + HO_2_, etc.) was estimated.

In other words, RO_2_ fate was tracked as a diagnostic variable but did not directly impact the product distribution (SILOX series, SV series, and concentration of VP). In addition to the adjustable parameters β and δ, a third adjustable parameter (λ, 0 – 1) was fit. The parameter λ proportionally reduced aerosol yields by multiplying the semivolatile concentrations from the gas phase solver prior to solving for partitioning (i.e., eq 1). The three adjustable parameters (β, δ, and λ) were estimated by exhaustive parameter search and minimization of objective function (eq 3) which includes equal contributions of mean error and mean bias in aerosol yield and mean normalized bias in the siloxanol number

$$f\left(\lambda ,\delta ,\beta \right)=\frac{Y_{MB}^{2}}{\max\left(Y_{MB}^{2}\right)}+\frac{Y_{ME}^{2}}{\max\left(Y_{ME}^{2}\right)}+\frac{{SN}_{MNB}^{2}}{\max\left({SN}_{MNB}^{2}\right)}$$ 4

where *Y* is yield, MB is mean bias, ME is mean error, SN is siloxanol number, and MNB is mean normalized bias. The definitions of each error statistic used in the paper are in the Supporting Information (eqs S4–S6).

## 3 Results and Discussion

This section first evaluates the influence of CO on particle formation and radical termination, followed by an analysis of particle phase products using UPLC-MS to assess how altering RO_2_ fate in a range of OH_exp_ shapes chemical composition. We then examine trends in SOA yield across OH_exp_, compare experimental and modeled results using both filter and filter plus PUF samples, and conclude with implications for D_5_ oxidation chemistry in relation to prior studies. New metrics for the siloxanol degree of substitution and siloxanol prevalence in the aerosol phase are used to facilitate model-observation comparison.

OFR experiments that included chemical composition analysis are given in Table 1. Results from these seven experiments are discussed throughout the Results and Discussion, with additional insight drawn from OFR experiments without chemical composition analysis (Table S1). These are supplemented by chemical composition analysis from ambient samples taken in New York City and chamber oxidation samples reported in L25.

### 3.1 Impact of CO on Particle Formation

To orient readers to typical results from the OFR system, detailed results for a pair of conditions are presented first. A key technique employed throughout this work is CO injection into the OFR to manipulate RO_2_ radical fate (favoring reaction with OH under no CO and favoring reaction with HO_2_ with CO present). Figure 1 presents a time series for a continuous OFR experiment where the only change was the presence or absence of CO injection (experiments 15 and 16, with IDs E10-CO and E10, respectively, in Table 1). Both experiments employed identical initial D_5_ concentrations and the OFR configurations. Conducting the experiments consecutively on the same day minimizes confounding effects from other variables (e.g., lamp intensity, tubing configuration, flow calibrations, and so on).

**Figure 1:**
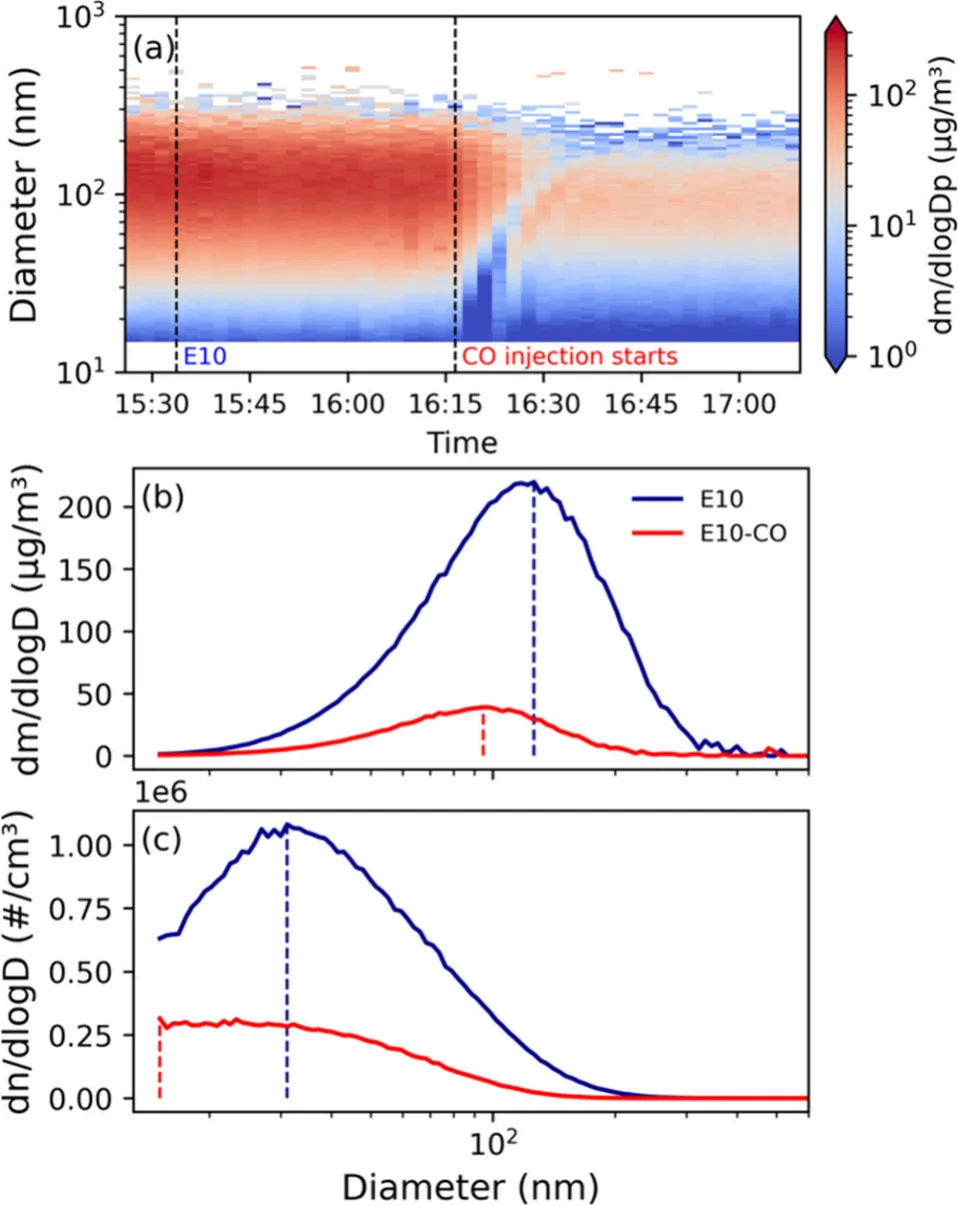
Effect of CO addition on particle size distribution during D_5_ oxidation. (a) Time-resolved SMPS contour plot of aerosol mass distribution as a function of particle diameter and time. The experiment began under CO-free conditions (E10); ∼65 ppm of CO (OHR_ext_ = 332 s^–1^) was introduced at 16:16, marking the transition to the E10-CO period. (b) Average mass distributions and (c) number distributions during E10 (blue) and E10-CO (red) periods, showing shifts in the particle mass and number modes following CO addition.

Using 65 ppm of CO to introduce external OH reactivity (OHR_ext_) promotes HO_2_ radical formation via the reaction CO + OH → CO_2_ + HO_2_, thereby elevating the HO_2_/OH ratio and biasing the radical termination regime toward RO_2_ + HO_2_.^42,48^ In these experimental conditions, the presence of CO lowered OH_exp_ from 3.2 × 10^12^ to 4.7 × 10^11^ molecules cm^–3^ s, decreasing the equivalent photochemical aging from 31 to 5 days. Integrated radical termination rates quantified the regime shift; in the absence of CO, RO_2_ + OH and RO_2_ + HO_2_ pathways contributed comparably (47% and 53%, respectively), while in the presence of CO, RO_2_ + HO_2_ dominated (95%).

CO addition substantially suppressed particle formation. Following CO injection, both aerosol mass and number concentrations decreased (Figure 1). The mass-mode diameter shifted from 126 nm (E10) to 95 nm (E10-CO), and the number-mode diameter shifted from 31 nm to the SMPS detection limit (15.1 nm). Total SOA mass concentration decreased from 140 μg m^–3^ (E10) to 19 μg m^–3^ (E10-CO), representing an 85% reduction. These observations show the substantial effect of CO injection on the system and the rapid response of the OFR to the chemical perturbation of the CO.

### 3.2 Oxidation Product Chemistry; Impact of OH_exp_ and RO_2_ Fate

To probe how varying OH_exp_ and RO_2_ termination regimes shape the distribution and quantity of D_5_ oxidation products, we examine trends across a suite of OFR experiments spanning a wide range of OH_exp_ (∼4 × 10^11^–9.4 × 10^12^ molecules cm^–3^ s; Table 1). Radical termination conditions were modulated via CO addition, altering the relative contribution of RO_2_ + OH versus RO_2_ + HO_2_ pathways.

Measurements of siloxanols and other oVMS are semiquantitative, meaning that concentrations are approximated using a surrogate standard, because standards are not yet commercially available. Ionization efficiency in the ESI(−) source may enhance some oVMS signals relative to others, so relative signals within a sample do not necessarily reflect relative abundance. The ESI(−) conditions applied are selective toward negatively ionizing functional groups. In their study of surrogate compounds, M24 reports much higher sensitivity of this method to silanols that more readily deprotonate (p*K*_a_s 10–15.4) than silylmethanol (p*K*_a_ 15.9). On the basis of this finding, this MS detection method is expected to respond more to siloxanols than less acidic functional groups with higher p*K*_a_s.^21^ Ionization efficiencies are compound-specific and consistent across samples, especially for oVMS having long retention times and separation from the sample matrix. Working within these limitations, our data analysis focuses on the relative changes across samples, including shifting oVMS concentrations and product distributions within and across gas and particle phases.

Figure 2 summarizes the particle phase oxidation product distributions for five OFR experiments conducted under comparable precursor loading conditions, with average initial D_5_ concentrations ([D_5_]_0_) of 107 ± 31 ppb (1σ). Products shown include the siloxanol products family (Si_5_), encompassing siloxanol through pentasiloxanol, along with dimers (Si_10_). Additional minor oxidation products, including silylmethanols, were detected at very low relative signal abundance but are not shown in the figure for clarity. Formate esters and ether hydroperoxides, that were previously observed in the gas phase,^48^ were not detected, with formate esters expected to hydrolyze to siloxanol in the presence of water^52^ and hydroperoxides prone to decomposition. Carboxylic acid products of D_5_ were also not observed, consistent with prior experimental and theoretical studies.^48,52,53^

**Figure 2:**
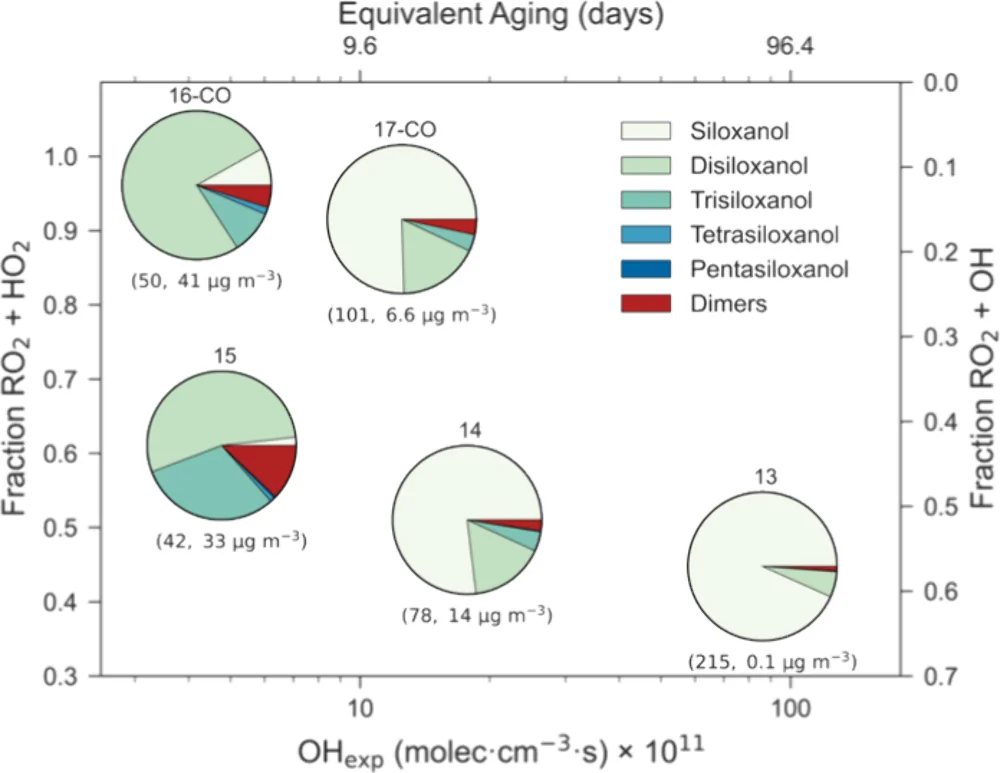
Relative distribution of particle phase D_5_ oxidation products for five OFR samples arranged in order of increasing OH_exp_ (left to right). Each pie chart illustrates the relative UPLC-MS signal of oVMS detected in QFF samples. The first numerical values are SMPS-derived aerosol mass concentration, and the second value is the semiquantified mass concentration collected on QFFs. The center of each pie chart corresponds to the OH exposure and RO_2_ fate of the associated experiment.

Under low OH_exp_, particle phase oxidation products extend beyond strictly first-generation siloxanols, indicating that D_5_ oxidation is not limited to a single functionalization step even under relatively mild oxidation. Under HO_2_-dominated conditions (e.g., 16-CO), the particle phase was enriched in early generation siloxanols, with disiloxanol comprising the dominant signal distribution (∼75%), followed by tetrasiloxanol and trace higher-generation siloxanols. Minor dimeric products (∼5%) contributed to the semiquantified signal (Figure S6c). Under similar OH_exp_ but with a decreased frequency of RO_2_ + HO_2_ reactions (e.g., 15), later-generation siloxanols and dimers grew in abundance relative to siloxanol and disiloxanol.

This paired comparison (16-CO vs 15) suggests that RO_2_ fate affects particle phase composition under low-to-moderate OH_exp_, with the enhanced RO_2_+OH-influenced experiment exhibiting increased relative contributions from later-generation siloxanols (e.g., trisiloxanol and pentasiloxanol) and high molecular weight products. As discussed below, this effect diminishes or disappears at higher OH_exp_ values. The cause of the difference in product distribution is not known, although previous theoretical and experimental studies offer insights. RO_2_ + HO_2_ reactions are predicted to form hydroperoxides; however, theoretical and experimental studies indicate that this pathway is kinetically limited for some cyclic siloxanes, and the resulting hydroperoxides are not observed or seen at low apparent yields experimentally.^48,54,55^

Calculated RO_2_ lifetimes are longest in experiment 15 (∼0.99 s), compared with ∼0.19 s in 16-CO and still shorter values in the remaining experiments (based on mean OH, HO_2_, and RO_2_ concentrations). These longer lifetimes are consistent with unimolecular RO_2_ isomerization pathways leading to polysiloxanol formation, as suggested by theoretical studies.^56^ However, reported RO_2_ isomerization rates appear too slow to fully account for the observed products. Future work should also consider the possibility that RO_2_ + OH generates RO + HO_2_, enabling RO mediated disiloxanol formation.

Differences in SMPS-derived mass between these low–OH_exp_ experiments fall within typical OFR variability and are not therefore interpreted as evidence for systematic SOA yield dependence on RO_2_ termination. At moderate OH_exp_ (∼1–2 × 10^12^ molecules cm^–3^ s), compositional differences between radical regimes diminish. Experiments 17-CO and 14 showed nearly identical UPLC-MS profiles dominated by siloxanol, disiloxanol, and trisiloxanol, while aerosol mass differences correlated with precursor concentration and C_OA_ rather than RO_2_ termination. Thus, at these intermediate exposures, OH_exp_ exerts a stronger control on aerosol mass than radical branching.

At the highest OH_exp_ (∼9.4 × 10^12^ molecules cm^–3^ s), the particle phase UPLC-MS signal became very low compared to the SMPS-detected particle volume. Within the comparatively small set of resolved compounds, siloxanol comprised >90% of the signal. Similar trends have been reported in prior OFR and chamber studies, where extreme OH_exp_ shifted oxidation chemistry toward gas-phase fragmentation, thereby shifting the detected oxidation product distribution away from multigeneration products and yielding chemically simplified particle phases dominated by more oxidation-resistant, low-volatility species.^57–59^ While siloxanol dominance is widely reported across chamber, OFR, and ambient studies,^13,21,24,28–30,55,60,61^ several experiments have also observed increased contribution from ring-opened and higher-molecular-weight products (C_15_ and above) at elevated OH_exp_.^21,26^

At high OH_exp_, the divergence between the declining UPLC-MS signal and continued SMPS mass growth is substantial. The semiquantified SOA mass (0.1 μg m^–3^) is more than 3 orders of magnitude lower than the SMPS-measured aerosol mass (215 μg m^–3^). This divergence indicates that an increasing fraction of total aerosol mass is not represented by the subset of products detectable by UPLC–MS, rather than indicating a suppression of oxidation chemistry. Several methodological factors may contribute to this difference, including (i) the assumption of uniform ionization efficiency and use of a single surrogate standard in semiquantitation; (ii) incomplete extraction of low-solubility, high molecular weight SOA products from QFFs; (iii) degradation of labile species during sample preparation; and (iv) formation of oligomeric or polymeric condensed-phase products at high OH_exp_ not in the targeted compound list. These discrepancies could be reduced with authentic oVMS standards that enable recovery studies and instrument calibration. Unlike many non-VMS precursors, which exhibit nonmonotonic and declining SOA formation trends with increasing OH_exp_ due to a balance between functionalization and fragmentation reactions and heterogeneous oxidation,^37,58,62^ prior siloxanes studies consistently report increasing SOA formation with OH_exp,_ ^25,29–34^ highlighting a key distinction between D_5_ oxidation chemistry and that of many traditional VOC precursors.

Building on the RO_2_ fate sensitivity observed at low OH_exp_, a siloxane-specific interpretation is that increasing oxidation intensity shortens radical lifetimes and shifts the detected particle phase product distribution toward early generation siloxanols, consistent with theoretical work showing that polysiloxanol formation can arise from unimolecular RO_2_ radical rearrangement/isomerization that competes with bimolecular termination.^53,54,56^ Under high OH_exp_, fast bimolecular reactions may outcompete these rearrangements and favor early generation products. In addition, at higher aerosol loadings, enhanced condensed phase processing (e.g., polymerization or oligomerization of more substituted polysiloxanols) could reduce their detectability by UPLC–MS and contribute to the apparent enrichment of simpler siloxanols.

Taken together, these findings highlight an apparent discrepancy between the chemically resolved particle phase products and the evolution of aerosol mass measured by SMPS with increasing OH_exp_. This behavior may reflect multiple, nonexclusive processes. Under high OH_exp_ conditions, the evolution of particle phase products detectable by UPLC–MS does not follow a classical, sequential multigenerational functionalization process, despite continued SOA mass growth. In addition, highly oxidized or oligomeric products may be under-recovered in UPLC–MS due to low ionization efficiency, incomplete extraction from QFFs, or decomposition during sample preparation. Increased particle phase volume at high OH_exp_ may further enhance oligomerization or other multiphase processes, leading to the formation of dimers and related products that are not detected by UPLC–MS.^29^ These species may contribute to SMPS mass but evade detection by UPLC–MS. The current data do not allow these contributions to be quantified separately.

### 3.3 Organic Carbon Analysis

Offline OC analysis of selected QFF samples tested mass closure vs SMPS mass and provided insights into the volatility and thermal stability of the particle phase products. Samples 15 and 16-CO were analyzed due to their lower OH_exp_ (about 4–5 days of aging) and high siloxanol concentrations. The OC-derived organic matter (OM) mass agreed reasonably with SMPS-derived aerosol mass for both samples (e.g., 281.9 μg OM vs 314.5 μg from SMPS for sample 15 and 350.7 μg OM vs 281.9 μg from SMPS for sample 16-CO), validating the consistency across measurement approaches.

Thermal desorption analysis showed that a substantial fraction of the OC evolved at 870 °C (66% for 15 and 78% for 16-CO), indicating the presence of low volatility and thermally stable compounds. Only minor fractions were desorbed at 310 °C (13% and 7%, respectively), a range typically associated with volatile or semivolatile compounds. These findings suggest formation of low-volatility SOA under OFR conditions, either intrinsically or through sampling artifacts on QFF, and/or additional transformations on the QFF filter during OC analysis (i.e., by addition of heat in an oxygen free environment). Low-volatility and thermally stable SOA from D_5_ has been also reported previously under OFR conditions.^26,33,34^

### 3.4 Chemical Box Model Simulations

To complement the experimental results, the K23 multigenerational aging framework (progressive oxidation of semivolatiles (SV) through a VBS with bin-hopping) was extended as described in the methods section. The main extension was explicit inclusion of siloxanols.^34^ The K23 multigenerational model (and its extended version implemented herein) is RO_2_ “unaware.” The adjustable parameters introduced in the extended version are adjusted until predictions are statistically optimal through the OFR data of this work; then, residuals are analyzed for association with RO_2_ fate and OH_exp_ to help disentangle the driving factors to degree of siloxanol substitution and yield.

The general characteristics of the multigenerational model are introduced first. SOA Yield vs OH_exp_ is shown in Figure 3, and ultimate yield at high OH_exp_ is sensitive to the fragmentation parameter (β). At high values of β, there is a peak yield at around 1.7 × 10^12^ molecules cm^–3^ s of OH_exp_. With no fragmentation the yield at high OH_exp_ goes to one.

**Figure 3:**
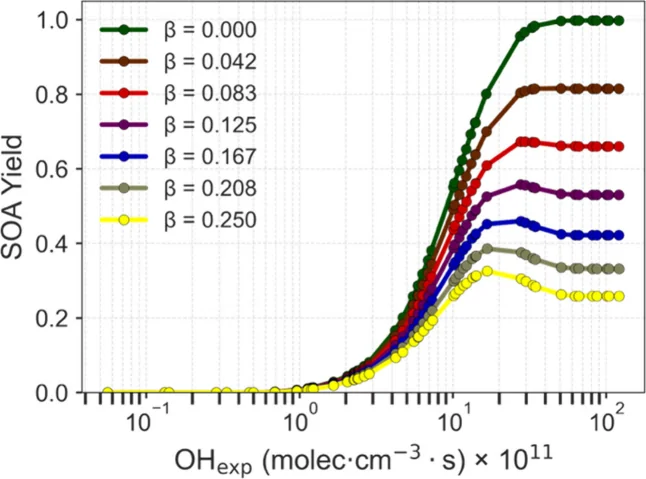
SOA Yields predicted by the extended multigenerational oxidation model under values of the fragmentation parameter β ranging from 0 to 0.25. All cases are for oxidation of 100 ppb of D_5_ and aerosol partitioning under absorption only.

The evolution of the semivolatile VBS bins is shown in Figure 4. It starts with a distribution of products (mainly c* = 10,000) as specified by the initial oxidation stoichiometric coefficients and then evolves to lower c* bins via bin hopping. The mole-weighted average SV bin (solid line, right-hand axis) starts at 1.24 and increases monotonically to 6. Thin lines with symbols show the average SV number predicted in the aerosol phase, via absorptive partitioning, under a range of organic aerosol loads. The average aerosol SV number is always greater than the aerosol plus gas value, with the difference being the greatest for low aerosol loads.

**Figure 4:**
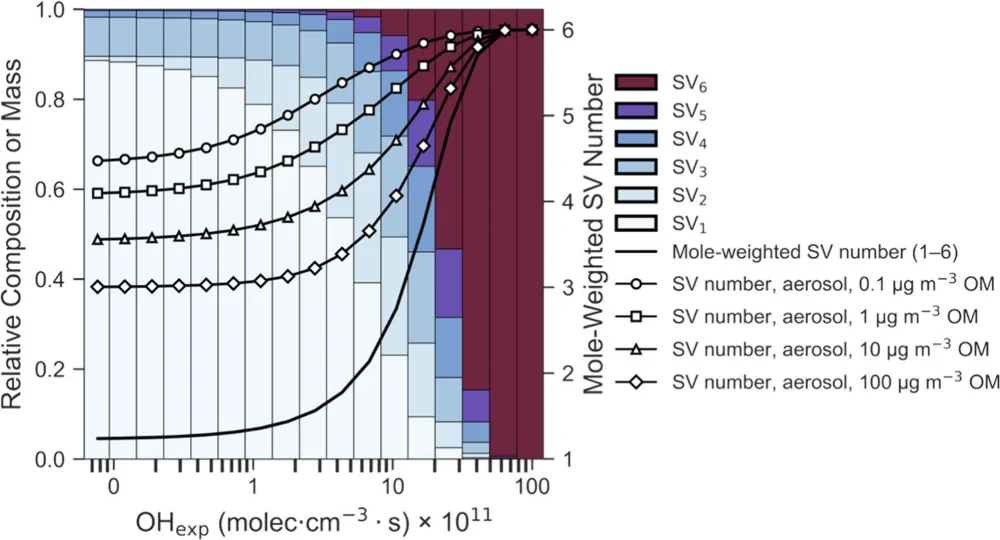
Semivolatile products (all phases) from the oxidation mechanism (δ=0.01). 100 ppb of D_5_ oxidized with multigenerational oxidation (SV_n_ + OH → SV_n+1_) with SV_6_ as a terminal product. The mean SV number (mole-weighted average bin number, all phases) increases from 1.24 to 6. Lines with markers show the mean SV number predicted in the aerosol phase via absorptive partitioning to 0.1, 1, 10, and 100 μg m^–3^ of aerosol, respectively.

The evolution of the siloxanol series is very similar and is shown in Figure 5. The initial oxidation yields only the siloxanol, and then additional reactions are required to create polysiloxanols, consistent with the mechanism proposed in L25.^28^ Similar to Figure 4, the siloxanol number (all phases) is shown in the solid line, and begins at one, and then increases monotonically. Under absorptive partitioning, the aerosol phase average siloxanol number is higher, with the divergence largest under low organic loadings. Because the mechanism has each siloxanol reaction with OH leading to the next siloxanol in the series, and to unspecified (chemically more complex) products that are only represented by the SV bin surrogate, the total yield of polysiloxanols peaks at an intermediate OH_exp_. This is sensitive to the exact values of β and δ.

**Figure 5:**
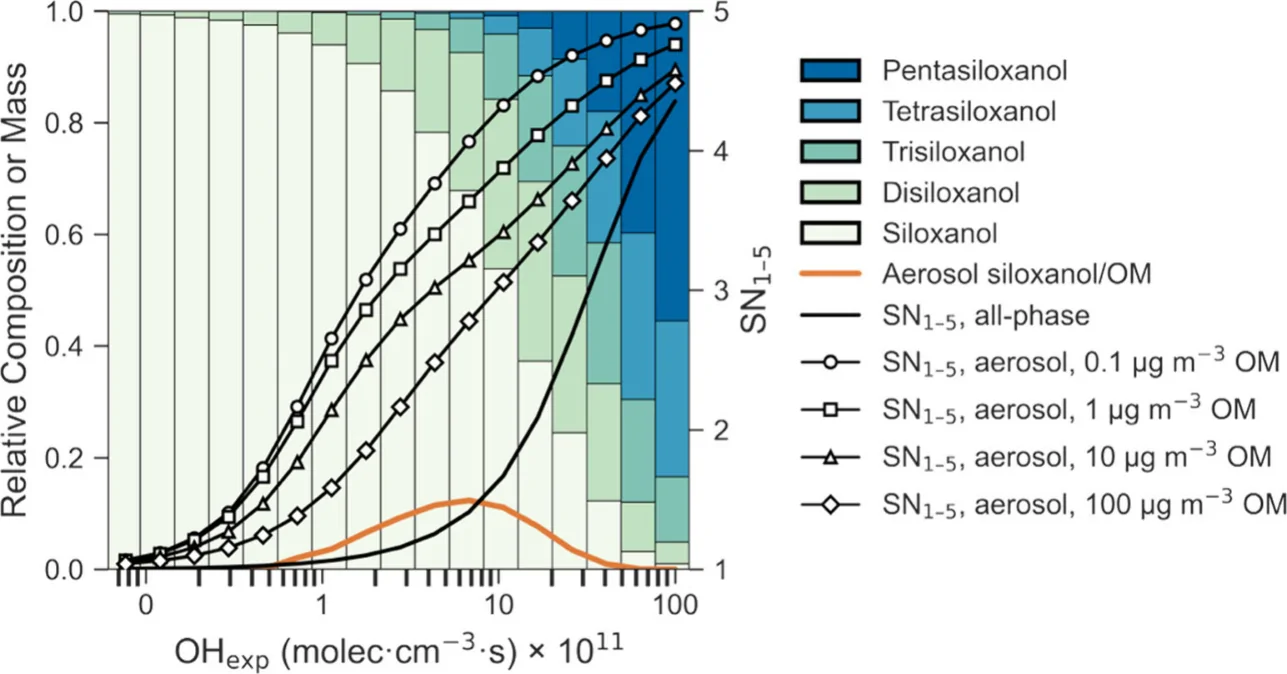
Siloxanols (all phases) produced from the oxidation mechanism (δ=0.5). 100 ppb of D_5_ oxidized with the degree of OH substitution increasing with OH_exp_. The mean siloxanol number of all phases (mole-weighted average number of OH per siloxane) increases from 1 to 5. The ratio of total particle phase siloxanols mass to organic matter mass peaks at around 0.12; model OM is initially dominated by SV surrogate compounds rather than siloxanols. The siloxanol number of the aerosol phase depends on available organic mass (OM) for absorptive partitioning, is always larger than the total number, decreases with OM, and is shown for OM values of 0.1, 1, 10, and 100 μg m^–3^.

The best combination of adjustable parameters, minimizing bias and error in the objective function (see methods), was λ ≈ 0.12, δ ≈ 0.09, and β ≈ 0, suggesting that fragmentation is not supported by our data, while siloxanol formation is required but at relatively low branching ratios. The model vs observation yield and siloxanol numbers are shown in Figure 6. While the model reproduces the average tendency for increased yield with increasing OH_exp_, the model’s inherent tendency for increase in the siloxanol number runs counter to a modest decrease in siloxanol number with OH_exp_ in the experimental data.

**Figure 6:**
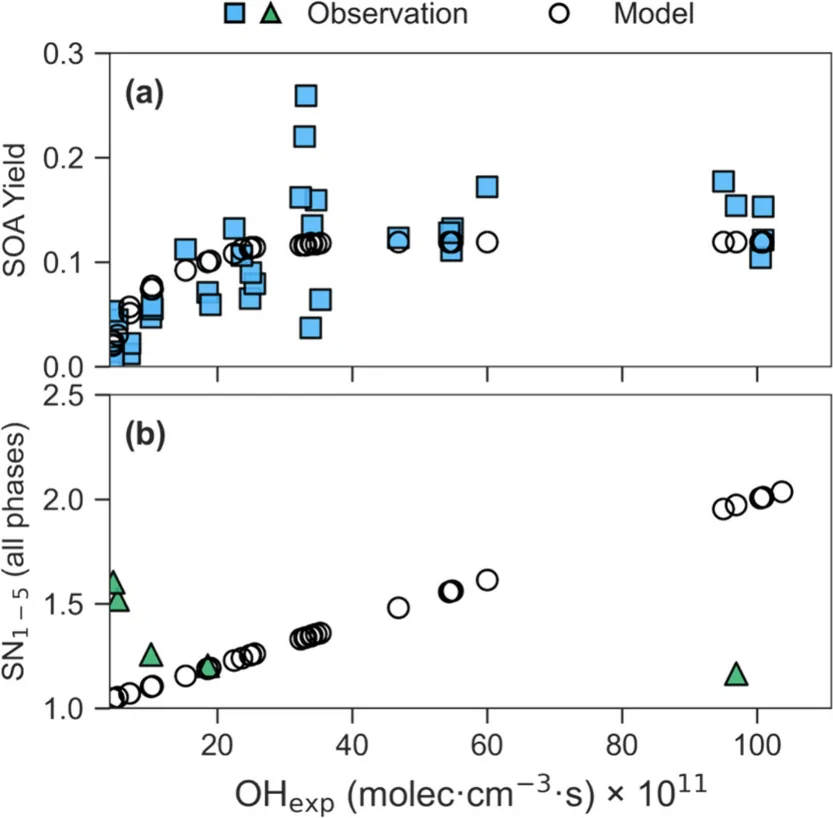
(a) Model and observation aerosol yield as a function of OH_exp_ from OFR experiments and (b) model and observation siloxanol number SN_1–5_ (see methods), also as a function of OH_exp_. Model parameters in both plots are λ=0.12, β=0, and δ=0.09.

Figure S12 shows the residuals of the model as a function of RO_2_ fate and OH_exp_ to assess whether there is statistically significant experimental evidence of an RO_2_ effect on yield or siloxanol number.

One clear result from the statistical comparison of model and experimental data is a divergence in the prediction and experiment for the degree of OH substitution in the siloxanols (Figure 6b), quantified by the siloxanol number. The modeled siloxanol number increased monotonically with OH_exp_, whereas experimental data showed a decrease, indicating a fundamental mismatch between sequential hydroxylation assumptions and the observed chemistry. This can be seen in Figure S12f.

A second, more tentative outcome from the statistical comparison concerns the influence of the RO_2_ fate on the aerosol yield. The evidence is subtle (borderline in statistical significance) but suggests that the RO_2_ + OH pathway may produce slightly lower volatility products and higher aerosol yields than the RO_2_ + HO_2_ pathway. This inference rests on the following pattern: the model (which treats RO_2_ fate only as a diagnostic variable) slightly overpredicted yields under RO_2_ + HO_2_-dominated conditions and slightly underpredicted yields when RO_2_ + OH was more prevalent. Reconciling this discrepancy would require either increasing the yield of low-volatility products from RO_2_ + OH reactions, decreasing such yields from RO_2_ + HO_2_ reactions, or a combination of both.

The statistical support for this effect is weak (p = 0.04 for the RO_2_ term), and the RO_2_ dependence does not persist in multiple regression, including both RO_2_ fate and OH_exp_ as predictors. Full regression details are provided in SI section S9. It should be noted that the pattern hinted at by the regression is consistent with the independent chemical composition results in Figure 2 at low OH_exp_, with enhanced disiloxanol, trisiloxanol, and dimer in sample 15, formed under a greater contribution of RO_2_ + OH. Nonetheless, these patterns merit follow-up studies using systematic variation of RO_2_ fate with techniques sensitive to volatility and molecular composition, such as thermal desorption mass spectrometry. These would likely confirm and clarify whether the RO_2_ fate exerts a measurable influence on product volatility and SOA yield.

### 3.5 Comparison of Ambient, Chamber, and OFR Observations with Model Predictions

Box model predictions are compared with OFR, chamber, and ambient observations across OH_exp_ in Figure 7 to evaluate siloxanol distributions and oxidation metrics, with focus on the mole-weighted siloxanol number SN_1–2_ calculated using siloxanol and disiloxanol only (eq 4). For OFR experiments and the model, the aerosol siloxanol/OM ratio was evaluated to assess the contribution of chemically resolved siloxanols to total organic aerosol mass constrained by SMPS. These comparisons were used to diagnose limitations in the model mechanism.

**Figure 7:**
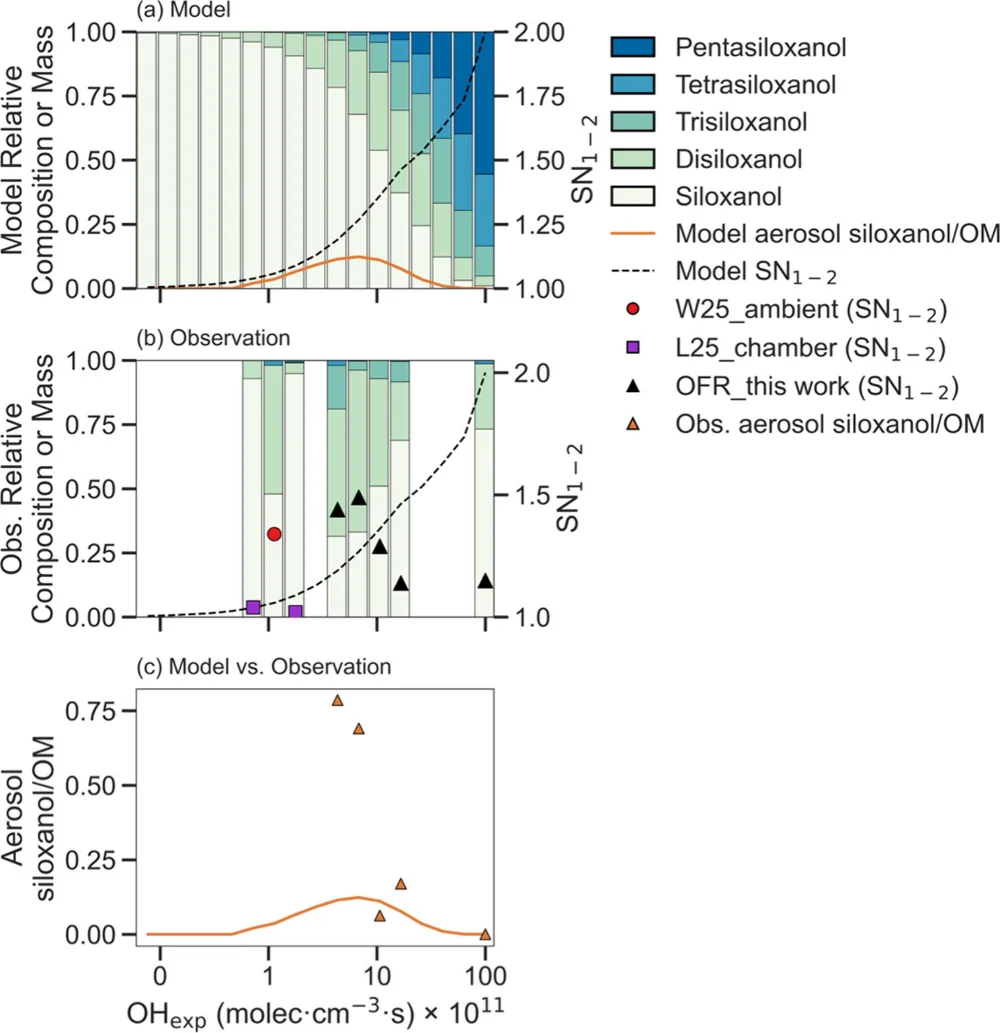
(a) Box-model predictions of all-phase siloxanol composition and mole-weighted siloxanol number as a function of OH_exp_ (reproduced from Figure 6). (b) Observed siloxanol distributions from OFR experiments in this work, chamber experiments (L25), and ambient measurements (W25), derived from UPLC–MS. All data sets are shown as relative mass fractions of major siloxanol species, while symbols indicate the observed mole-weighted siloxanol number considering only siloxanol and disiloxanol (1–2); the dashed line shows the corresponding box-model prediction. (c) Comparison of modeled and observed (OFR) aerosol-phase siloxanol mass fraction (siloxanol/OM).

The multigenerational oxidation framework predicts a progressive shift toward higher-generation siloxanols with an increasing OH_exp_ reflected in a monotonic increase in the modeled siloxanol number (Figure 7a). This behavior arises from the assumption of sequential hydroxylation in which each OH reaction advances siloxanols to the next substitution level. Observed siloxanol distributions across ambient, chamber, and OFR data sets are summarized in Figure 7b.

Ambient observations from the W25 data set provide a benchmark for siloxanol formation under chemically complex urban conditions.^23^ In New York City, oxidation occurs under high-NO_*x*_ conditions; furthermore, both OH and Cl radicals are likely present. Chamber studies indicate that OH- and Cl-initiated oxidation of VMS proceeds via similar pathways and produces comparable product distributions.^13,31,52^ Under high-NO_x_ conditions typical of urban environments, RO_2_ radicals are expected to react predominantly with NO, forming alkoxy radicals that can access unimolecular rearrangements not represented in conventional non-VMS VOC mechanisms, leading to products such as formate esters.^53,56^

In the ambient average (W25, details for the seven samples shown in Figure S7), the mole-weighted SN_1–2_ of near 1.4, with a similar value of SN_1–5_, indicates that higher-generation siloxanols contribute negligibly to the chemically resolved oxidation metric and that the all-phase siloxanol burden is dominated by siloxanol and disiloxanol. This behavior is also consistent with prior NYC work showing that siloxanol is predominantly in the gas phase during summer,^23^ so that all-phase composition remains weighted toward the more volatile early generation products even when particle phase uptake occurs. The selective appearance of tetrasiloxanol without measurable trisiloxanol is inconsistent with a stepwise addition of siloxanol functional groups and instead supports our earlier interpretation that siloxanol substitution may reflect branching among oxidation channels and/or positional “hopping” of reactive intermediates, rather than a fixed stepwise progression that must populate every intermediate at detectable levels. To facilitate comparisons, an effective average ambient OH_exp_ for the W25 sampling period was estimated using a VOC clock approach (SI Section S11).^63–65^ At the same estimated OH_exp_, the model underpredicts the degree of oxidation observed in the W25 ambient samples. Whereas the model remains dominated by siloxanol, yielding a siloxanol number near unity, the ambient samples exhibit a substantially higher siloxanol number driven by enhanced disiloxanol contribution. This difference may reflect uncertainty in the estimated OH_exp_ for the NYC sample or additional reaction pathways not represented in the model mechanism. Notably, even when the upper bound of the estimated OH_exp_ (∼1.6 × 10^11^ molecules cm^–3^ s; 95th percentile) is considered, the model-observation divergence persists. This discrepancy indicates that, under urban conditions, the model underestimates product diversity, even when higher-generation siloxanols contribute negligibly to the oxidation metric.

Environmental chamber observations (L25) provide a controlled framework for isolating how seed aerosol surface area, composition, and phase state govern the partitioning of siloxanol oxidation products into the particle phase.^28^ The all-phase siloxanol distribution is strongly dominated by siloxanol in both unseeded (OH_exp_ of 7 × 10^10^ molecules cm^–3^ s) and AS seeded (average OH_exp_ of 1.5 × 10^11^ molecules cm^–3^ s) conditions, indicating that the bulk chemical outcome of D_5_ oxidation at these relatively low OH_exp_ remains centered on early generation products. In the unseeded sample, siloxanol accounts for >90% of the resolved siloxanol signal with disiloxanol at low abundance and no detectable tri–pentasiloxanols, yielding a siloxanol number near unity (SN_1–5_ ≈ SN_1–2_ ≈ 1). In the seeded experiments, the all-phase distribution shifts only modestly, where siloxanol remains dominant (>90%) while small but measurable amounts of higher-generation siloxanols appear (tetra > tri > pentasiloxanol), yet the siloxanol number remains ∼1 (SN_1–5_ ≈ SN_1–2_). The near-identity of SN_1–5_ and SN_1–2_ in both seeded and unseeded experiments indicates that tri through pentasiloxanol contribute negligibly to the aggregate oxidation metric, even when detectable. This behavior is consistent with AS seed aerosol providing surface area and condensed-phase mass that facilitates adsorption and absorption of a fraction of the oxidation products,^23,27,28^ where here it reveals small amounts of higher-generation species without substantially altering the integrated all-phase siloxanol number. At comparable OH_exp_, the box model broadly reproduces the L25 chamber results, though it slightly overpredicts SN_1–2_.

As shown in Figure 7b, five OFR observations exhibit a nonmonotonic response where higher-generation siloxanols (di through tetra) are most apparent at intermediate OH_exp_. In contrast, the UPLC-MS resolved siloxanols in observations become dominated by early generation products, primarily siloxanol and disiloxanol, at high OH_exp_. This is also reflected in the siloxanol numbers: SN_1–5_ exceeds SN_1–2_ primarily at low OH_exp_, while the two metrics converge at higher OH_exp_, indicating that increasing oxidative exposure does not translate into continued advancement toward higher substitution within the detectable product pool observed by UPLC-MS. A similar trend is observed for particle phase oxidation products (Section 3.2) and contrasts with the monotonic progression predicted by the multigenerational model (Figure 7a). Despite very different oxidation histories, the NYC ambient sample and the highest–OH_exp_ OFR experiment exhibit remarkably similar detectable siloxanol speciation, dominated by siloxanol and disiloxanols with trace tetrasiloxanol and trisiloxanol not contributing appreciably to the detectable signal. This convergence suggests that atmospheric chemical context, rather than cumulative OH_exp_ alone, controls siloxanol formation and persistence.

This divergence in the model prediction and OFR observations is further emphasized by the particle phase siloxanol mass fraction (siloxanol/OM; Figure 7c). In the multigenerational model, siloxanol/OM is near zero at low OH_exp_, increases to a maximum (∼0.1) at intermediate OH_exp_, and then declines again at high OH_exp_. In contrast, the OFR experiments exhibit much higher maximum siloxanol/OM (up to ∼0.8), followed by a similar monotonic decline with increasing OH_exp_ that converges toward zero at high OH_exp_. Both the model and observations support increasing prevalence of nonsiloxanol compounds in the particle phase at high OH_exp_. However, at moderate OH_exp_, the divergence is striking. The observations indicate a dominant contribution of the siloxanol and disiloxanol to the aerosol, whereas the model predicts these species to be minor contributors, with unspecified surrogate compounds dominating instead. Together, these results demonstrate that the model’s assumption of continuous multigenerational hydroxylation fails to reproduce the observed evolution of siloxanol number and relative signal abundance.

This discrepancy suggests missing chemical processes, additional loss pathways, branching chemistry, instability of higher-generation siloxanols, and/or progressive transfer of oxidation products into highly oxidized, oligomeric, or fragmented species that are not captured within the targeted UPLC–MS-resolved product set under high OH_exp_ conditions (Section 3.2). One such limitation is that only OH-initiated polysiloxanol formation is in the mechanism ([Disp-formula eqR4]), while unimolecular (autoxidation) channels^28,48,56^ are not. Based on the unimolecular isomerization rate of 8 × 10^–3^ s^–1^, this pathway was estimated to be minor under our OFR conditions, though this rate is poorly constrained. Future work will require improved experimental constraints and revised modeling frameworks to accurately represent siloxanol formation and fate.

Taken together, this work's findings show that SOA formation from D_5_ is primarily governed by oxidation extent, while RO_2_ radical termination pathways influence particle phase chemical composition at low OH_exp_ without governing overall SOA mass. As OH_exp_ increases, SOA mass continues to grow even as the abundance of chemically resolved oxidation products, including siloxanols measured by UPLC–MS, declines. This suggests that higher-generation siloxanols are not dominant conserved oxidation end points and that particles likely include many products that are not recovered or are insensitive to the UPLC–MS methods used here. Model–measurement comparisons show that the extended K23 multigenerational framework reproduces the overall SOA yield trend with OH_exp_ but systematically overpredicts siloxanol persistence, indicating that sequential functionalization alone cannot explain the observed evolution of product distributions.

Accurate representation of D_5_ oxidation requires a mechanistic understanding of the formation of its major initial product, the siloxanol, which provides the foundation for describing the subsequent generations of products. The multigenerational mechanism will require consideration of a wider range of options beyond sequential functionalization, including branching, instability, radical rearrangement, and competitive radical pathways, particularly under conditions relevant to the OFR and atmospheric cases. Future studies integrating molecular-level speciation with mechanistically expanded models will be needed to constrain these processes across laboratory and atmospheric oxidation regimes.

## Supplementary Material



## Notes

OFR experimental setup and schematic; summary of OFR experiments; gas-phase D5 sampling and GC-MS/MS analytical methods, including calibration procedures, instrument parameters, and quantitation equations; D5-derived SOA density determination by gravimetric analysis; aerosol sample collection and UPLC-MS/MS analytical methods; semiquantified distributions of aerosol- and gas-phase oxidation products from OFR experiments and ambient NYC samples; impurity analysis and attribution of oD4 and oD6 oxidation products; evaluation of filter sampling artifacts using tandem quartz fiber filters; kinetic box model reactions, parameters, and statistical evaluation methods; OFR UV lamp photon flux characterization; and ambient NYC OH exposure estimation using the chemical clock method (PDF)
