# Supplementary material for: Decamethylcyclopentasiloxane (D5) Oxidation: Product Chemistry, Influence of RO2 Fate, and Secondary Aerosol Production
Source: ACS EST Air. 2026 Jul 10;3(8):1988–2000. doi: 10.1021/acsestair.6c00037 (PMC13488271; doi:10.1021/acsestair.6c00037)
Supplement: Supplementary file 1 [file ea6c00037_si_001.pdf]

Supporting Information of

Decamethylcyclopentasiloxane (D5) oxidation: product chemistry, influence of RO_2 fate, and secondary aerosol production

Saeideh Mohammadi[1,2], Jeewani N. Meepage[3], Christopher E. Brunet[2,4], Rachel F. Marek[2,4], Keri C. Hornbuckle[2,4], Eleanor C. Browne[5], Elizabeth A. Stone*[1,3], Charles O. Stanier*[1,2]

[1]Department of Chemical and Biochemical Engineering, University of Iowa, Iowa City, 52242, USA.
[2]IIHR-Hydroscience and Engineering, University of Iowa, Iowa City, Iowa 52242, USA.
[3]Department of Chemistry, University of Iowa, Iowa City, Iowa 52242, USA.
[4]Department of Civil and Environmental Engineering, University of Iowa, Iowa City, 52242, USA.
[5]Department of Chemistry and Cooperative Institute for Research in Environmental Sciences, University of Colorado, Boulder, Colorado 80309, USA.

**Correspondence to*: Charles O. Stanier (charles-stanier@uiowa.edu) and Elizabeth A. Stone (betsy-stone@uiowa.edu)

This file includes:

Number of pages: 25

Number of figures: 15

Number of tables: 10

S1. OFR Experimental Setup

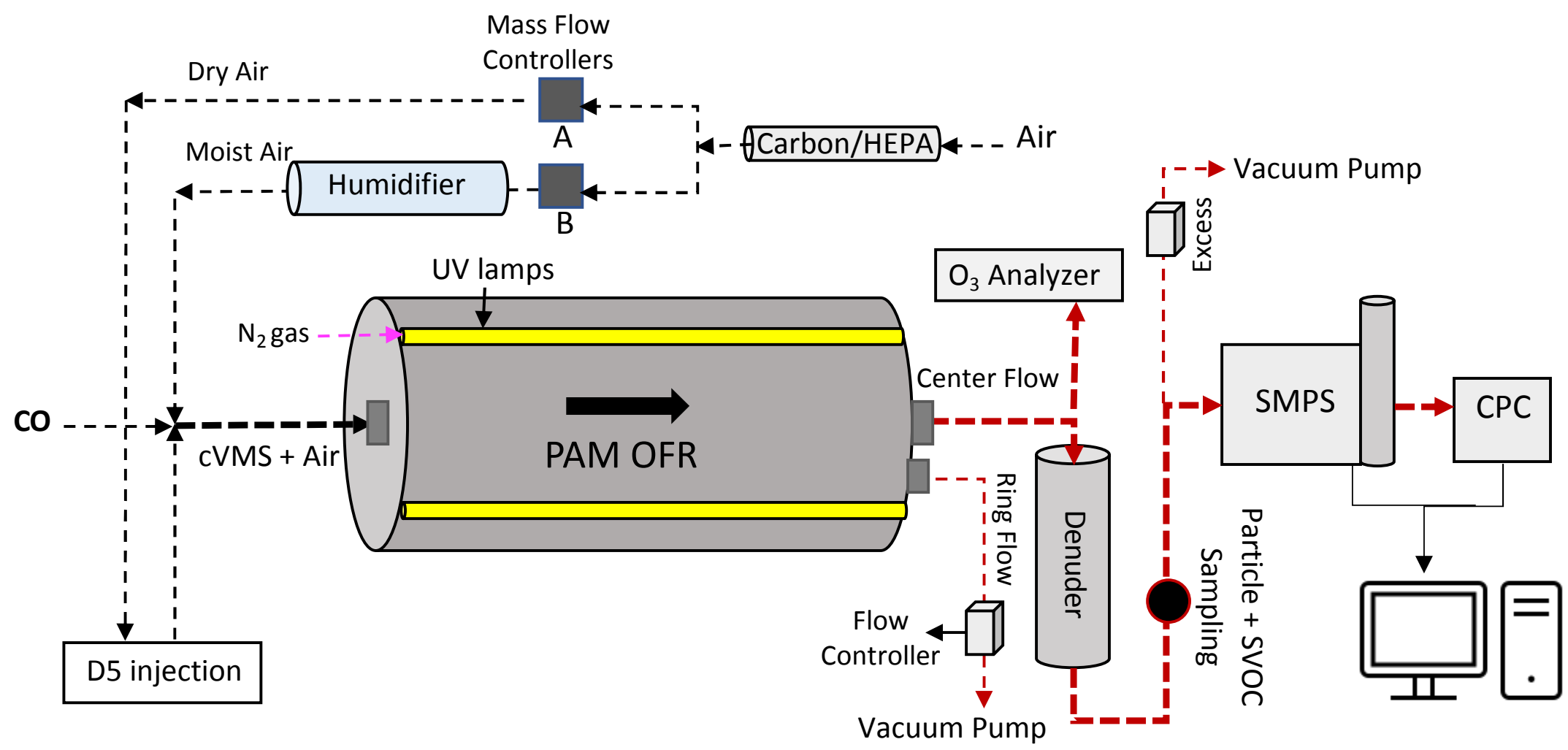

Figure S1: Schematic of the PAM OFR system coupled to an O_3 analyzer, SMPS, and CPC. Black dashed lines indicate OFR inlet tubing; red dashed lines show outlet tubing, and pink dashed lines represent nitrogen gas inlets for UV light cooling. Air is filtered through a HEPA filter to remove particles and a carbon filter to eliminate VOCs, followed by a humidifier to regulate humidity for OH generation. Diluted VMS standards are injected using a syringe pump. Downstream O_3 is monitored and removed via a Carulite-equipped denuder. SOA concentration is measured with SMPS and CPC for yield assessment.

Constructed from anodized aluminum, the OFR is a horizontal cylinder (46 cm length × 22 cm inner diameter; ~13.3 L volume). Four low-pressure mercury lamps (HSM-UNV-214-LT5 ballast) surrounded the chamber and emitted both 185 nm and 254 nm ultraviolet (UV) light, generating OH radicals through the photolysis of water vapor (H_2O) and molecular oxygen (O_2). The lamps were enclosed in quartz sleeves and actively cooled with a nitrogen curtain to stabilize operating temperatures and protect the quartz from UV degradation.

Table S1: Details of all OFR experiments conducted in this study, including relative humidity (RH), normalized lamp power (see section S11), CO additions, external OH reactivity (OHR_{ext}), OH concentration, OH exposure (OH_{exp} × 10^{11} molec cm^{-3} s), equivalent atmospheric aging, HO_2/OH ratios, RO_2 termination pathways (RO_2 + HO_2 vs. RO_2 + OH), reacted D5 and SOA mass concentrations, with corresponding sampling configuration and analysis (QFF, PUF, and OC).

Exp.	RH	Lamp 185 power	Lamp 254 power	CO (ppm)	OHR_{ext} (s^{-1})	[OH] ×10^9 (cm^{-3})	OH_{exp}×10^{11} (s cm^{-3})	Eq. Aging (days)	HO_2/OH	RO_2+ HO_2	RO_2+ OH	ΔD5 (μg m^{-3})	SOA Mass (μg m^{-3})	Samples
1	0.05	0.24	0.92	43	200	2.99	4.63	4	96.67	0.94	0.06	945	50 ± 2	16-CO (QFF/PUF/OC)
2	0.04	0.04	0.36	0	0	3.40	5.26	5	11.29	0.65	0.34	938	42 ± 2	15 (QFF/PUF/OC)
3	0.1	0.38	0.38	22	100	6.65	10.31	10	54.46	0.91	0.09	2182	101 ± 2	17-CO (QFF1/QFF2/PUF)
4	0.05	0.13	0.92	0	0	12.20	18.91	18	9.16	0.56	0.44	1104	78 ± 3	14 (QFF/PUF)
5	0.23	0.99	0.99	0	0	60.80	94.24	91	10.61	0.47	0.53	1411	215 ± 5	13 (QFF/PUF)
6	0.1	0.38	0.38	0	0	16.34	25.33	24	16.27	0.67	0.33	2721	242 ± 4	
7	0.08	0.38	0.97	65	300	4.65	7.21	7	97.65	0.94	0.06	2157	48 ± 1	
8	0.08	0.38	0.97	0	0	22.35	34.64	33	11.84	0.60	0.39	3254	119 ± 2	
9	0.12	0.86	0.99	43	200	15.39	23.85	23	47.10	0.91	0.09	2221	232 ± 4	
10	0.12	0.86	0.99	0	0	38.01	58.91	57	13.34	0.56	0.44	2231	381 ± 4	
11	0.05	0.99	0.99	0	0	21.84	33.86	33	18.38	0.65	0.35	1260	199 ± 3	
12	0.08	0.99	0.99	0	0	29.55	45.80	44	16.19	0.60	0.39	1643	201 ± 2	
13	0.1	0.99	0.99	0	0	34.53	53.52	52	15.08	0.60	0.40	2789	354 ± 1	
14	0.05	0.24	0.92	0	0	14.49	22.45	22	11.68	0.59	0.40	1113	146 ± 2	
15	0.08	0.24	0.92	65	300	3.04	4.71	5	121.50	0.95	0.05	1427	19 ± 1	
16	0.08	0.24	0.92	0	0	20.71	32.09	31	9.56	0.53	0.47	1457	140 ± 3	
17	0.24	0.99	0.99	0	0	62.91	97.52	94	10.32	0.44	0.56	624	94 ± 2	

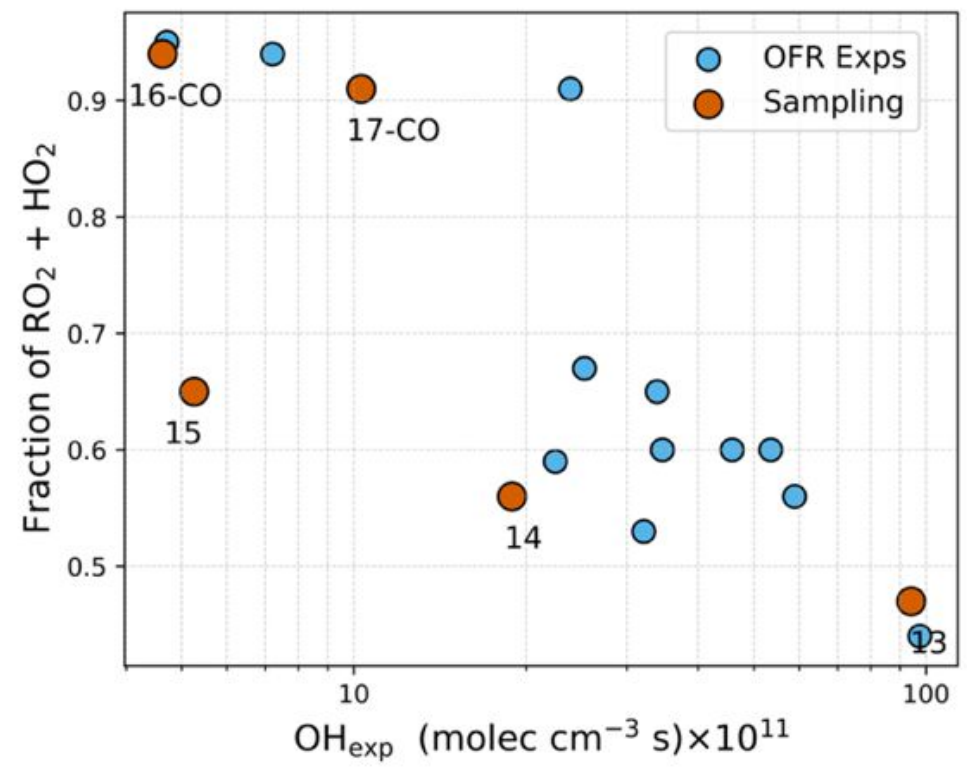

Figure S2: OFR experiments represented in Table S1 are shown as fraction of $RO_2 + HO_2$ versus OH_{exp} (molec cm^{-3} s × 10^{11}). Experiments that included gas- and particle phase sampling of D5 oxidation products using QFF and PUF are indicated by red circles, and labels indicate the corresponding sample IDs.

S2. Gas D5 sampling and Offline Analysis

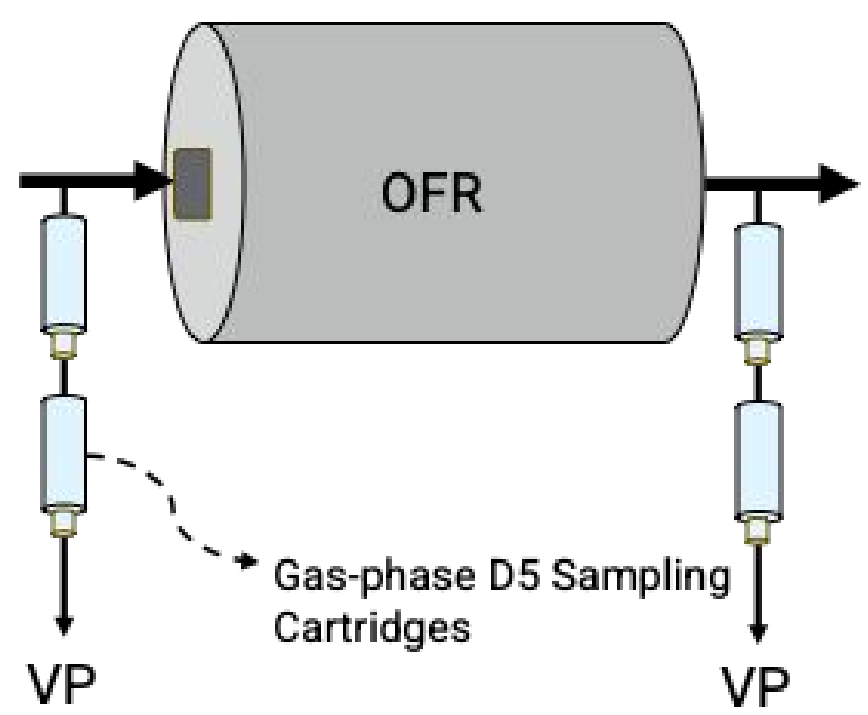

Figure S3. D5 gas collection setup with two SPE cartridges (ISOLUTE ENV+) for GC-MS analysis.

Accurate quantification of precursor concentrations and unreacted species in the OFR was achieved by collecting gas phase samples upstream and downstream of the reactor. Samples were collected using ISOLUTE ENV+ cartridges (Biotage, 915-0001-A) arranged sequentially to capture potential breakthrough, with field blanks included for quality control. For sample analysis, a calibration standard (90 ng ml^{-1} D3, 90 ng ml^{-1} D4, 90 ng ml^{-1} D5, 90 ng ml^{-1} D6, 94.5 ng ml^{-1} L5, 98.28 ng ml^{-1} L7, 127 ng ml^{-1} 13C-D3, 125 ng ml^{-1} 13C-D4, 123 ng ml^{-1} 13C-D5, 113 ng ml^{-1} 13C-D6, 35 ng ml^{-1} PCB-30), surrogate standard solution (127 ng ml^{-1} 13C-D3, 125 ng ml^{-1} 13C-D4, 123 ng ml^{-1} 13C-D5, 113 ng ml^{-1} 13C-D6) and internal standard solution (500 ng ml^{-1} PCB-30) were prepared in hexane via serial dilution. All samples and blanks were spiked with 0.1 ml of the surrogate standard solution, extracted with 1.5 ml of hexane, and then spiked with 0.1 ml of the internal standard solution. A reference solution was prepared with each run by spiking 1.5 ml of hexane with identical masses of the surrogate standards and internal standards. Extracts, calibration standards and reference solutions were analyzed using an Agilent 7250 GC/Q-TOF system operating in MS/MS mode.[1]

The MS/MS method for the detection of target compounds, surrogate standards, and the internal standard was developed as follows. ~100 ng ml^{-1} standards of the stock solutions were prepared volumetrically in hexane and analyzed in full scan mode (Table S2). The most abundant ion for each compound was selected as the precursor ion, and a mixed standard of all chemicals was prepared and analyzed to determine an oven gradient that achieved full separation of each compound and their associated retention

times. A series of product ion scan methods were developed using the oven gradient and precursor ions determined above. The collision energies for each precursor ion were tested between 5 eV and 60 eV in increments of 5 eV. The most abundant product ion-collision energy pairing was selected as the quantitative ion for each compound, and the second largest was selected as the qualitative ion. Based on these tests, the parameters of the MS/MS method were finalized. A series of method blanks and serial dilutions of the mixed standards were analyzed using this method to determine the method detection limit and the linear range.

Table S2: Chemicals used in analysis of gas phase cartridges

Chemical	Abbrev.	Purpose	Purity	Manufacturer	Catalog Number
Hexamethylcyclotrisiloxane	D3	Target	0.98	Cambridge Isotope Laboratories Inc (CIL). Andover, MA	ULM-9687-S
Octamethylcyclotetrasiloxane	D4	Target	0.98	CIL	ULM-9441-MT-S
Decamethylcyclopentasiloxane	D5	Target	0.97	CIL	ULM-9442-MT-S
Dodecamethylcyclohexasiloxane	D6	Target	98%,	CIL	CLM-10232-S
Dodecamethylpentasiloxane	L5	Target	0.97	Sigma-Aldrich. St. Louis, MO	447269
Hexadecamethylheptasiloxane	L7	Target	0.97	AmBeed. Arlington Heights, IL	A207631
Hexamethylcyclotrisiloxane-13C6	13C-D3	Surrogate	0.98	CIL	CLM-9542-S
Octamethylcyclotetrasiloxane-13C8	13C-D4	Surrogate	0.98	CIL	CLM-9436-MT-S
Decamethylcyclopentasiloxane-13C10	13C-D5	Surrogate	0.98	CIL	CLM-9437-MT-S
Dodecamethylcyclohexasiloxane-13C6	13C-D6	Surrogate	0.98	CIL	CLM-10232-S
2,4,6-tricholorbiphenyl	PCB-30	Internal Standard	0.98	CIL	PCB-30
Hexane	Hexane	Solvent	Pesticide Grade	Fisher Chemical Pittsburgh, PA	H300-4

The GC-QTOF was equipped with an Agilent HP-5ms ultra inert column (30 m × 0.250 mm × 0.25 µm) with UHP helium as the carrier gas (constant flow 0.8 mL min^{-1}) and quench gas (4 mL min^{-1}) and UHP nitrogen as the collision gas (1 mL min^{-1}). The injection volume was 5 µL. The GC inlet operated at the following conditions: initial temperature 45 °C, initial time 0.06 min, ramp 600 °C min^{-1} to inlet temperature 200 °C. The GC oven temperature program was 45 °C for 5 min, 45 to 100 °C at 40 °C min^{-1} and hold for 0.5 min, 100 to 280 °C at 12 °C min^{-1} and hold for 3 min (total run time: 26 min). The MS transfer line temperature was held at 280 °C. Precursor ions, product ions, retention times, and collision energies for target analytes are shown below in Table S3.

Table S3: Analysis parameters for MS/MS method developed for the 7250 GC-QTOF.

Target Compound	Ion Type	Retention Time (min)	Precursor Ion (m/z)	Product Ion (m/z)	Collision Energy (eV)
13C-D3	Quant	6.5	212.05	195	50
13C-D4	Quant	8.5	288.07	76.05	40
13C-D5	Quant	10.3	364.1	76.05	35
13C-D6	Quant	12.3	345.03	75.04	25
D3	Quant	6.5	207.03	191	25

D4	Quant	8.5	281.05	73.04	35
D5	Quant	10.3	355.07	73.04	35
D6	Quant	12.3	341.02	73.04	25
L5	Quant	11.4	281.05	73.04	40
L7	Quant	14.9	221.08	73.04	30
PCB-30	Quant	16.96	257.9	186.02	35
13C-D3	Qual	6.5	212.05	76.05	25
13C-D4	Qual	8.5	288.07	254	30
13C-D5	Qual	10.3	364.1	266.99	25
13C-D6	Qual	12.3	345.03	324.9	15
D3	Qual	6.5	207.03	73.04	60
D4	Qual	8.5	281.05	248.98	30
D5	Qual	10.3	355.07	266.99	15
D6	Qual	12.3	341.02	324.9	15
L5	Qual	11.4	281.05	248.98	40
L7	Qual	14.9	221.08	221.08	10

Each analytical run contained four calibration standards and two of the reference solutions prepared above, and analytical blanks consisting of pure hexane were run between each set of standards and the samples. All samples were analyzed in duplicate. Following analysis, compound peak areas were quantified in Mass Hunter Qualitative Analysis 10.0. The relative response factor (RRF) was calculated for each target compound and the 13C surrogate standards relative to the PCB30 internal standard according to Eq. S1. RRFs were calculated for each analyte for four calibration standard injections using Eq. S1 and then averaged. Analyte masses in samples were then calculated using the average run RRF, the analyte peak areas, and the internal standard mass according to Eq. S2.

$$RRF = \frac{M_{c,C} * A_{is,C}}{M_{is,C} * A_{c,C}} \qquad \text{Eq. S1}$$

$$M_{c,S} = \frac{A_{c,S}}{A_{is,S}} * RRF * M_{is,S} \qquad \text{Eq. S2}$$

Where $M_{c,C}$ is the known mass of the target compound in the calibration standard.
$A_{is,C}$ is the measured peak area of the internal standard in the calibration standard.
$M_{is,C}$ is the known mass of the internal standard in the calibration standard.
$A_{c,C}$ is measured peak area of the target compound in the calibration standard.
$M_{c,S}$ is calculated mass of the target compound in the sample.
$A_{c,S}$ is measured peak area of the target compound in the sample.
$A_{is,S}$ is measured peak area of the internal standard in the sample.
$M_{is,S}$ is known mass of the internal standard in the sample.

S3. D5-derived SOA density

SOA mass was quantified gravimetrically in three OFR experiments using 47 mm Teflon filters with a pore size of 2.0 µm (Pall Life Sciences). Filters were weighed under controlled temperature and humidity conditions using a microbalance with 0.001 mg precision (Mettler Toledo XP26), following standard conditioning and static-neutralization procedures. Gravimetric SOA mass was compared with aerosol mass derived from SMPS size distributions, assuming a particle density of 1.2 g cm^{-3}. The close

agreement between gravimetric and SMPS-derived masses shown in Figure S4 supports the use of 1.2 g cm^{-3} as a representative density for D5-derived SOA in this work.

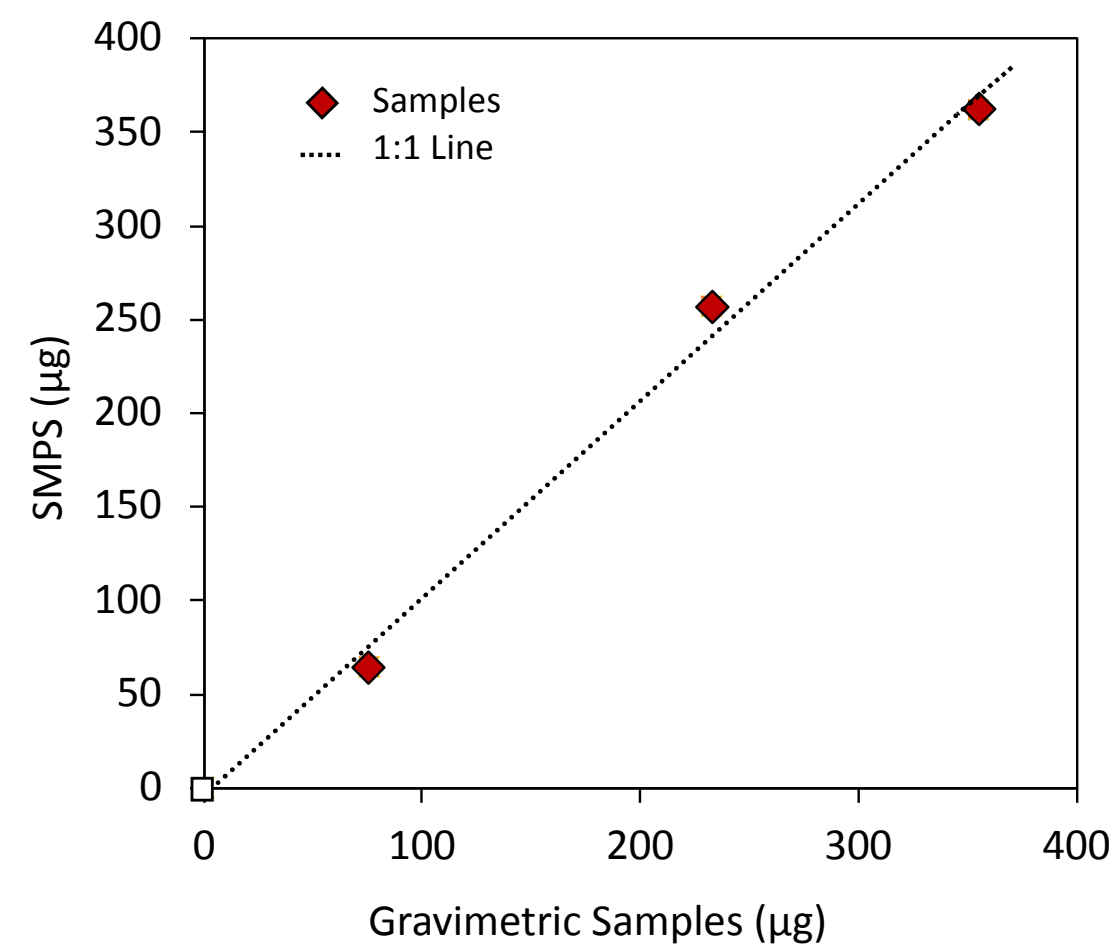

Figure S4. The comparison of gravimetric sampling data with SMPS-derived aerosol mass concentrations for three samples of D5-derived SOA collected downstream of the OFR. The gravimetric results demonstrated strong agreement with the SMPS values, aligning closely with the 1:1 reference line.

S4. D5-derived Aerosol Sample Collection and Analysis

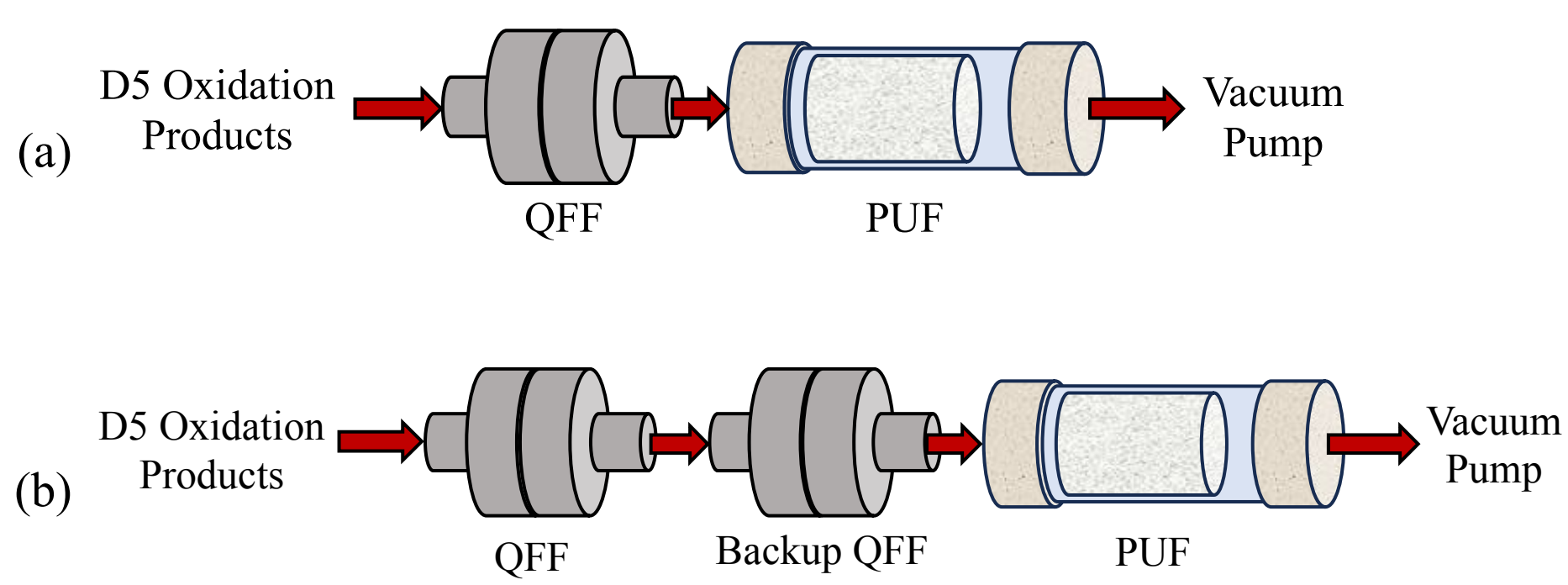

Figure S5. (a) Schematic of particle and gas samples from D5 oxidation collected on 90 mm quartz fiber filter (QFF, Tissuquartz, Pall Life Sciences) and polyurethane foam (PUF) filters at a constant flow rate downstream of the OFR and (b) with backup QFF added to the line for artifact analysis in experiment 17-CO.

S4.1. Ambient NYC Sample Information

Table S4: Summary of ambient samples collected during the NYC-METS 2022 campaign and used in this work. Samples were collected at the ASRC site in Manhattan, New York City, using co-located QFF and PUF substrates during alternating 12-h daytime (D) and nighttime (N) periods. Sampling procedures and chemical analysis are described in Welker et al. (2025).[2]

Label	W25 Sampling ID	Sampling start time	Sampling end time
July 25th (N)	AERO_NYC_PM 2.5_035 (N)	7/25/22 21:04	7/26/22 8:18
July 26th (D)	AERO_NYC_PM 2.5_036 (D)	7/26/22 8:54	7/26/22 20:13

July 26th (N)	AERO_NYC_PM 2.5_037 (N)	7/26/22 20:53	7/27/22 8:13
July 27th (D)	AERO_NYC_PM 2.5_038 (D)	7/27/22 8:49	7/27/22 20:10
July 27th (N)	AERO_NYC_PM 2.5_039 (N)	7/27/22 20:42	7/28/22 8:13
July 28th (D)	AERO_NYC_PM 2.5_040 (D)	7/28/22 8:59	7/28/22 20:12
July 28th (N)	AERO_NYC_PM 2.5_041 (N)	7/28/22 20:49	7/29/22 8:11

S4.2. Extraction of QFF and PUF

QFF samples were extracted following Meepage et al. (2024) using 95:5 (v/v) acetonitrile and ultrapure water, filtered to remove particulates, spiked with phenol-D5 (98%, Sigma-Aldrich, 1000 µg L^{-1}) as an internal standard, and concentrated to a final volume of 200 µL.[3]

PUF samples were extracted following Welker et al. (2025) using acetonitrile[2], concentrated to 1 mL using rotary evaporation (200 mbar, 120 rpm), filtered (0.2 µm), and reduced to 100 µL under a gentle stream of ultra-pure nitrogen at 30 °C using a microscale nitrogen evaporation system (ReactiTherm III TS 18824 and Reacti-Vap I 18825, Thermo Scientific).

S4.3. Chemical Analysis by Liquid Chromatography with Mass Spectrometry

For analysis of oxidized VMS products, an ultraperformance liquid chromatography–tandem mass spectrometry (UPLC-MS/MS) system was equipped with a reversed-phase BEH C-18 column (2.1 × 100 mm, 1.7 µm particle size) and a high-resolution Q-Exactive Quadrupole Orbitrap mass spectrometer as described by Meepage et al (2024).[3] The mass spectrometer operated in negative ion mode with a heated electrospray ionization source. A gradient elution method was used to ensure efficient separation of the target analytes, with acetonitrile-water solvent system, and a 10 mM ammonium bicarbonate-ammonium hydroxide buffer (pH 10). This analytical method is sensitivity for hydroxylated siloxanes. However, it was not effective in ionizing certain functional groups, such as silyl methanol and ether peroxides.

S4.4. Post Processing and Semi-Quantitation

Raw data were acquired using Xcalibur software (Thermo Scientific) and processed with Compound Discoverer 3.3.0 (Thermo Scientific). Signals detected in field blanks were background subtracted. Then, molecular formulas were assigned using high-resolution accurate mass (±5 ppm) and isotope patterns as described by Meepage et al. (2024).[3] Semi-quantitation was performed using TraceFinder v4.0 (Thermo Scientific). Direct quantification was not feasible, due to the lack of authentic standards, so a semi-quantitative approach was applied using tris(tert-butoxy) silanol as a surrogate standard (99.999%, Sigma-Aldrich). The standard curve gradients were set at 4, 38, 379, 946, 1893, 3785 nmol L^{-1} for tris(tert-butoxy) silanol and the linearity of the calibration (R^2) curve was calculated to >0.995. D5-phenol served as an internal standard to ensure consistent analytical performance across all samples.

Table S5: Summary of D5 oxidation products detected in filter samples from OFR experiments.

Type of Molecule		Calculated *m/z* [M-H]$^{-1}$	Calculated exact mass [M]	Assigned formula [M]	Retention time (s) (t_R)
D5-derived	Siloxanol	371.0654	372.0732	$C_9H_{28}O_6Si_5$	10.9, 11.1
	Disiloxanol	373.0447	374.0525	$C_8H_{26}O_7Si_5$	7.2, 7.5,7.6 7.9, 8.0
	Trisiloxanol	375.0239	376.0317	$C_7H_{24}O_8Si_5$	4.6, 5.0
	Tetrasiloxanol	377.0032	378.011	$C_6H_{22}O_9Si_5$	3
	Pentasiloxanol	378.9834	379.9912	$C_5H_{20}O_{10}Si_5$	1
	Silyl Methanol	385.0811	386.0889	$C_{10}H_{30}O_6Si_5$	11.9
	Silyl methanol with Siloxanol	387.0603	388.0681	$C_9H_{28}O_7Si_5$	6.4,7.6,8.3
	Dimers	731.0659	732.0737	$C_{15}H_{48}O_{14}Si_{10}$	15.5,16.2
		733.0451	734.0529	$C_{14}H_{46}O_{15}Si_{10}$	11.3
		735.0244	736.0322	$C_{13}H_{44}O_{16}Si_{10}$	10
		433.0114	434.0192	$C_8H_{26}O_9Si_6$	8.4, 8.6
		523.0615	524.0693	$C_{11}H_{36}O_{10}Si_7$	9.8, 10.2
		509.0095	510.0173	$C_9H_{30}O_{11}Si_7$	8.4
D4-derived	Siloxanols	297.0466	298.0544	$C_7H_{22}O_5Si_4$	8.6
		299.0259	300.0337	$C_6H_{20}O_6Si_4$	4.1, 4.4, 4.5, 4.6, 5.1, 5.3
		301.0051	302.013	$C_5H_{18}O_7Si_4$	1.4, 2.1
		302.9844	303.9922	$C_4H_{16}O_8Si_4$	0.8
D6-derived	Siloxanols	445.0842	446.092	$C_{11}H_{34}O_7Si_6$	13.3,13.6
		447.0634	448.0713	$C_{10}H_{32}O_8Si_6$	11.9, 11.0, 10.4, 10.3, 10.0, 9.9, 9.4, 9.0, 8.9,8.4,8.2,5.9
		449.0427	450.05053	$C_9H_{30}O_9Si_6$	7.2, 7.6, 7.7, 8.1
		451.022	452.0298	$C_8H_{28}O_{10}Si_6$	5.8

S4.5. Organic Carbon Analysis

Organic carbon (OC) and elemental carbon (EC) were quantified on 1.0 cm^2 punches of particulate matter collected on QFF using a thermal-optical analyzer (Sunset Laboratory Inc.), following the NIOSH 5040 method with a maximum temperature in the He stage at 870 ºC.[2] Organic matter (OM) values were calculated from measured OC using a compound-specific OM/OC ratio determined from the molecular

formula of the main oxidation product in this study (disiloxanol; $C_8H_{26}O_7Si_5$) to account for the contributions of all elements in the molecule.

S5. D5-derived Aerosol distribution and Semi-quantification

For D5, up to five sequential oxidation steps were observed, each corresponding to the replacement of a methyl group with a hydroxyl group. This yielded a homologous series of siloxanols, substituted siloxanol species, and multiple classes of dimeric oxidation products were identified, including products derived from D4 and D6. Full molecular assignments and analytical details are provided in Table S5.

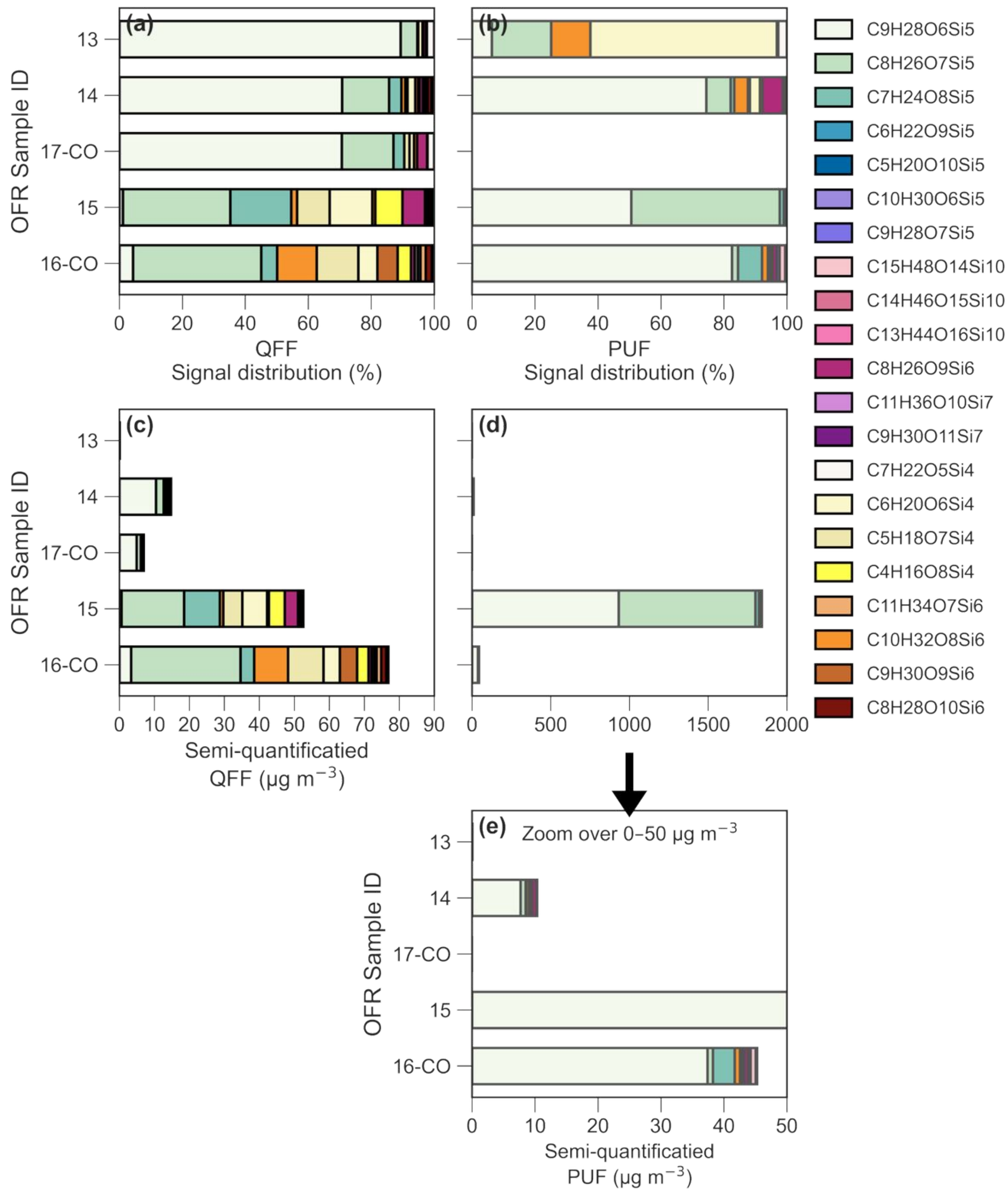

Figure S6: Signal distributions of all targeted D5 oxidation products collected on quartz fiber filters (QFF) and polyurethane foam (PUF) cartridges during selected OFR experiments and analyzed by UPLC–MS are shown in panels (a) and (b), respectively. Semi-quantified concentrations for QFF and PUF samples are shown in panels (c) and (d), respectively, with panel (e) showing a zoomed x-axis view of panel (d) (0–50 μg m^{-3}) to resolve high-abundance products observed in sample 15 and to improve comparability across samples. In all panels, OFR samples are ordered from low to high OH exposure (bottom to top).

In ambient aerosol samples collected in New York City (Table S4), D5 oxidation products were dominated by siloxanol and disiloxanol species, with tetrasiloxanol detected at lower abundance in a subset of samples, as shown in Figure S7.

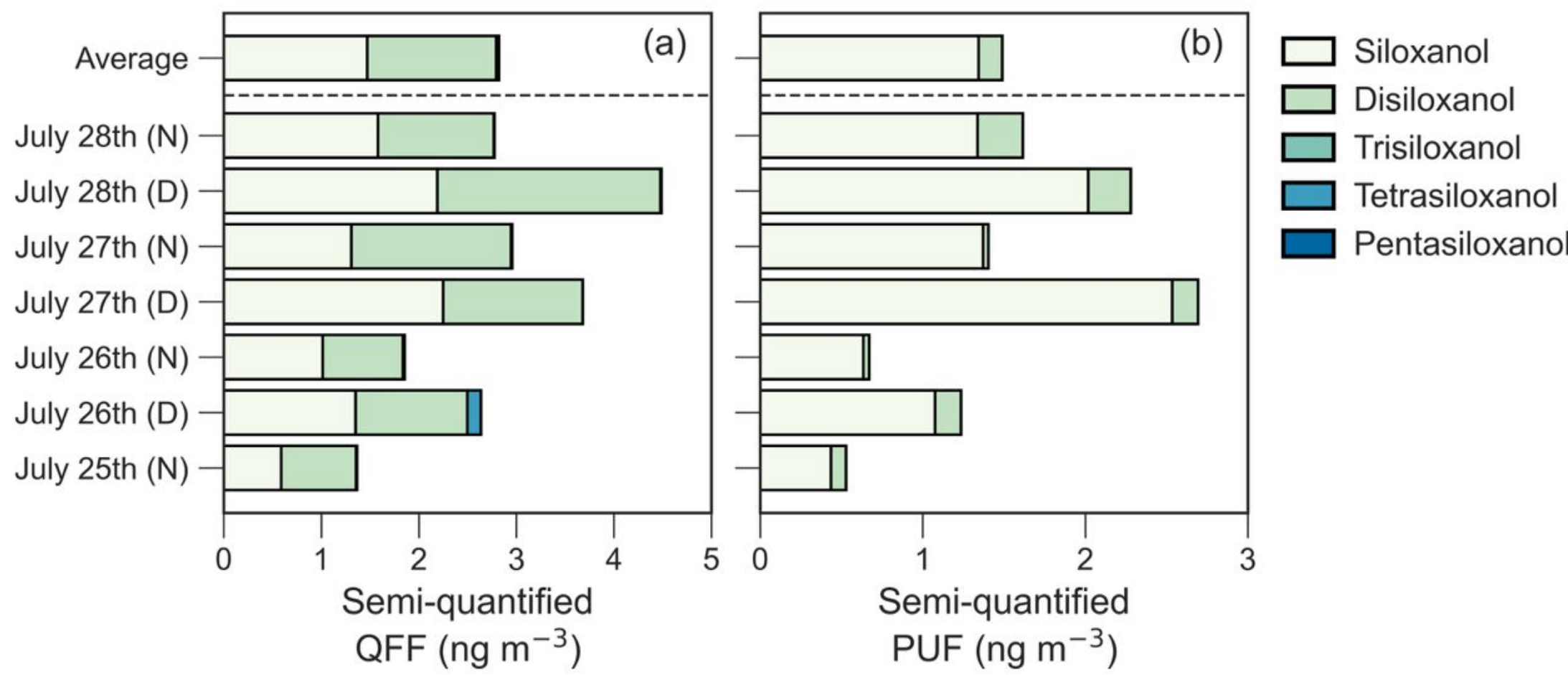

Figure S7: Semi-quantified concentrations of D5 oxidation products in seven ambient samples collected in New York City during the NYC-METS 2022 campaign[2], with the average of all samples shown at the top of each panel. Panels show semi-quantified concentrations (ng m^{-3}) on QFF (a) and PUF (b).

S6. Gas phase Oxidation Products Chemistry

Figure S8The relative distribution of oxidation products exhibited pronounced variability across PUF samples collected from the OFR, reflecting differences in radical chemistry and partitioning behavior. In 16-CO, the PUF phase was dominated by siloxanol, with notable but smaller contributions from trisiloxanol and disiloxanol, alongside minor amounts of dimers. Sample 15 displayed a markedly different profile, in which siloxanol and disiloxanol together accounted for the vast majority of the relative signal, accompanied by smaller proportions of trisiloxanol, and tetrasiloxanol. In contrast, sample 14 presented a simpler product spectrum dominated by siloxanol with secondary contributions from disiloxanol and trace dimers, while 13 exhibited minimal products in the PUF sample. Notably, no signal was observed in the PUF sample for 17-CO, which may be attributable to the inclusion of a backup QFF filter upstream of the PUF, as shown in figure S4. This configuration could have acted as a sorptive medium for semi-volatile species, effectively reducing their transfer to the PUF and potentially enhancing the positive artifact on the corresponding QFF.

In Figure S6, semi-quantitative estimates indicated that certain species, particularly in sample 15, reached very high apparent concentrations, with both siloxanol and disiloxanol in the hundreds of µg m^{-3} range, while sample 16-CO exhibited siloxanol in the tens of µg m^{-3} and other products in the low single-digit range. However, these absolute concentration values should be interpreted with caution due to the inherent uncertainties in semi-quantification. Potential error sources include variability in ionization efficiency across compound classes, matrix effects in the PUF extracts, and the absence of authentic standards for many detected products. Such factors can introduce substantial bias and uncertainty, particularly for comparing small differences between samples.

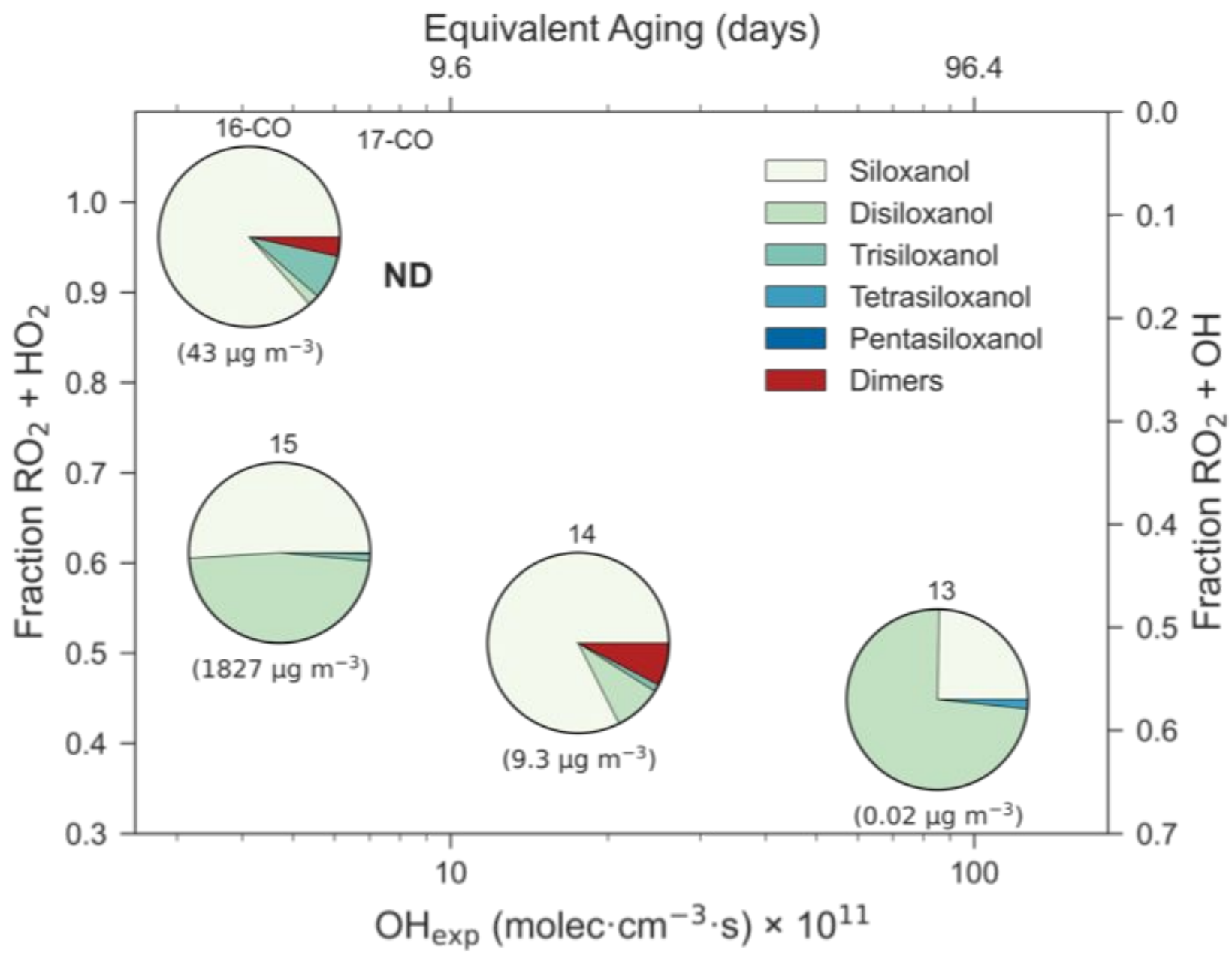

Figure S8. Relative distribution of gas phase D5 oxidation products for five OFR samples arranged in order of increasing OH_{exp} (left to right). Pie charts show the relative composition of semiquantified gas phase products collected on the PUF, from siloxanol through pentasiloxanol, and dimeric products. Minor products, including silylmethanol-containing compounds, were detected at low relative signal abundance, but are omitted for clarity. "ND" marks the 17-CO sample with no detectable gas phase products above method quantification limits, with a backup QFF used. Values beneath each pie correspond to the total semiquantified gas phase concentrations in PUF samples.

S7. Impurities in D5 Oxidation Experiments

In OFR sampling experiments, D5 was the intended precursor, but GC–MS/MS of the stock (MRM mode; standards D3–D6, L5, L7) demonstrated impurities D4 and D6 were present; both were also observed in selected OFR gas samples, confirming they entered the reactor. In the direct dilution of the D5 solution, D6 was quantified at approximately 5% and D4 at ~0.1% of the D5 mass. Subsequent analysis of some of the select OFR gas-D5 samples also confirmed the presence of D4 and D6 above the instrument detection limits. The relative abundances of these species in the OFR samples differed from those in the D5 solution, with D4 present at a higher proportion and D6 at a lower proportion relative to D5, likely due to the higher volatility of D4. Based on these measurements, it is estimated that for every 100 µg of D5 introduced, approximately 2 µg each of D4 and D6 were co-injected. The confirmed presence of these impurities in the injected material provides a basis for the formation of corresponding oxidation products. detected in QFF and PUF samples.

Because D4 and D6 were unintentionally introduced into the reactor, we therefore parsed products by silicon-backbone count, which is preserved in early-generation VMS oxidation (ring opening and hydroxylation/oxidation) and only changes under less favored scission that removes an entire Si–O unit. oD5 denotes D5-derived products (siloxanols, dimers, etc.), oD4 denotes products retaining an Si4 backbone, and oD6 denotes Si6 products. Assignment to oD4 and oD6 is also supported by the sample-to-sample co-variation between oD4/oD6 signals and the co-injected impurity fraction rather than with D5 consumption alone. The stacked bars in Figure S9 visualize the D4:D5:D6 distribution where the left bar estimates the injected composition (D5 solution included 2% D4 and 2% D6), while the QFF and PUF

bars (middle and right) show normalized particle phase products, revealing that oD4 and oD6 can contribute non-negligibly despite their minor presence at injection. Potential misassignment from in-source fragmentation was minimized by chromatographic separation. Additionally, the differing oDx distributions on QFF versus PUF are consistent with expected volatility/partitioning rather than backbone conversion. Collectively, these constraints support the interpretation that oD4 and oD6 observed in the particle phase arise from oxidation of D4 and D6 impurities co-injected with D5, not from D5 fragmentation into Si4/Si6 frameworks.

The inclusion of non-D5 species in the UPLC-MS analysis highlights the importance of precursor purity when interpreting oxidation products. While D5 served as the primary precursor, trace impurities such as D4 and D6 were also subject to oxidation and could contribute appreciably to the observed particle phase chemistry.

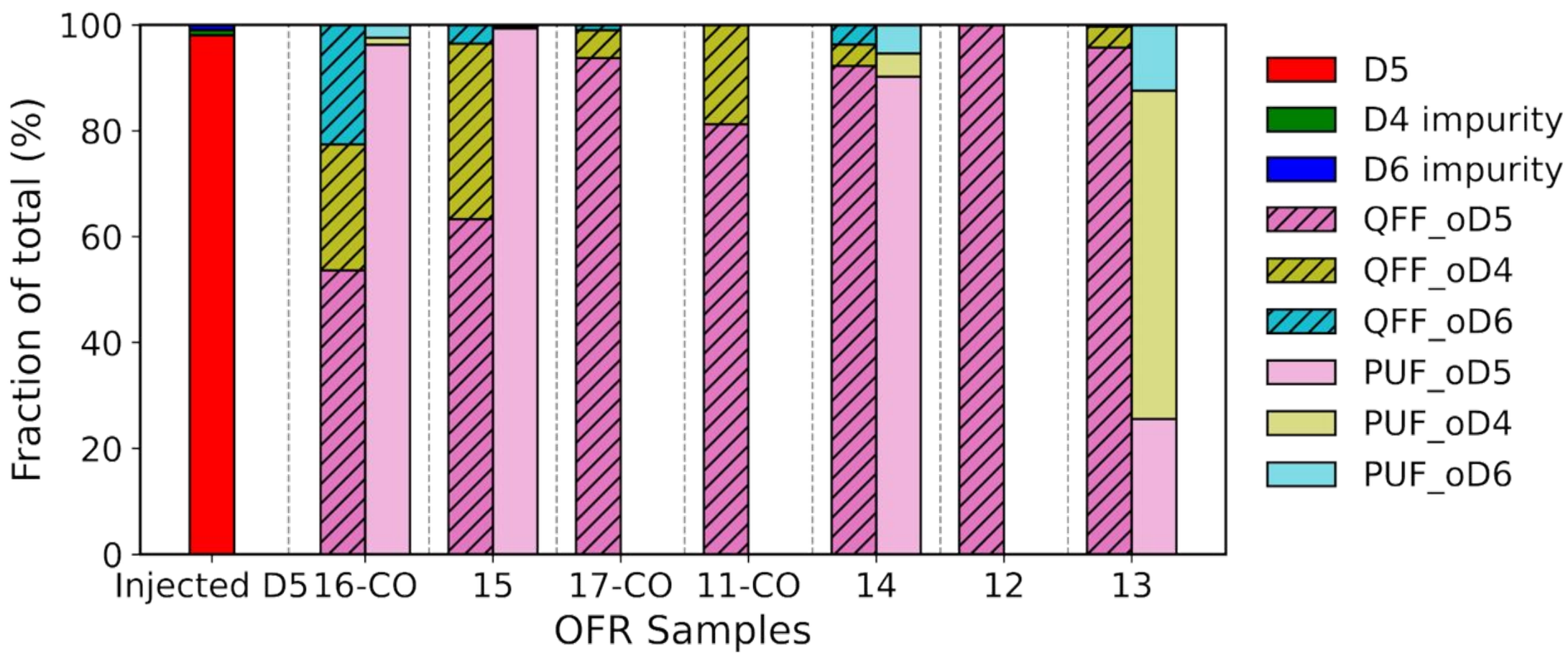

Figure S9. Composition of the impurity of injected gas D5 and results in its oxidation products for each OFR experiment. The first bar ("Injected D5") shows the intended precursor D5 (red) with co-injected impurities D4 (green) and D6 (blue), each fixed at 2 % of the D5 mass based on stock-solution characterization. For each OFR sample, QFF and PUF bars show normalized particle- and gas-phase oxidation products. Hatched QFF bars represent oD5, oD4, and oD6 products, while lighter-colored PUF bars indicate gas phase contributions. Vertical dashed lines separate individual OFR experiments.

S8. Filter Artifacts Evaluation Using Tandem QFFs

To evaluate potential positive artifacts arising from gas phase oxidation products adsorbing onto QFFs, a tandem filter configuration was implemented for sample 17-CO. In this setup (Figure S5b), a second QFF (backup filter) was placed downstream of the front filter to assess the extent of gas phase contamination that could be misattributed to the particle phase. Positive filter sampling artifacts were calculated as the percent of oxidized compounds on backup QFF relative to front QFF. To determine the particle phase mass, the amount of gas phase oxidation products adsorbed onto the front QFF was subtracted from the total measured on the front QFF. The oxidation product mass in the gas phase was determined as the sum of the gas phase collected in the PUF and the adsorbed gas phase mass on the front QFF. Although the PUF contained no detectable signal in this experiment, measurable concentrations on the backup QFF indicate that positive artifacts on the front filter were present. As shown in Figure S10, the extent of this artifact was compound dependent: the backup represented a minor fraction of the front filter mass for

siloxanol (~4%), but a substantially larger fraction for disiloxanol (~83%) and dimers (~40%). These results suggest that, while the bulk particle phase mass was dominated by condensed phase products, individual species can be strongly affected by gas phase adsorption onto the front filter. Accordingly, artifact-corrected values were applied to sample 17-CO only, whereas values from experiments lacking a backup QFF are reported as uncorrected. Based on the observations from ambient sampling in New York City, Welker et al. (2025) reported high positive artifacts for siloxanol (54–100%, avg ≈ 86%) and smaller artifacts for more oxidized siloxanols (≈10% for disiloxanol; negligible for tetrasiloxanols).[2] Compared to those ambient results, the magnitudes observed in our OFR experiment were generally lower for siloxanol and higher for disiloxanol and dimers. This difference reflects contrasts between ambient and OFR conditions, including mixture complexity, oxidation environment, and environmental parameters, as well as compound-dependent adsorption to the filter media under reactor conditions, noting that this assessment is based on a single experiment with a backup filter.

To account for sampling artifacts, the concentration associated with particle phase material was determined by removing the contribution measured on the backup QFF (QFF_2) from that on the front QFF (QFF_1) in sample 17-CO. This method relies on the assumption that gas phase adsorption occurs equally on both filters, implying that adsorption primarily reflects interactions with the filter substrate rather than the collected particulate mass. The relative magnitude of the positive sampling artifact on the front filter was determined using (Eq. S3).

$$Positive\ Artifact\ (\%) = \left(\frac{QFF_2}{QFF_1}\right) \times 100 \qquad \text{(Eq. S3)}$$

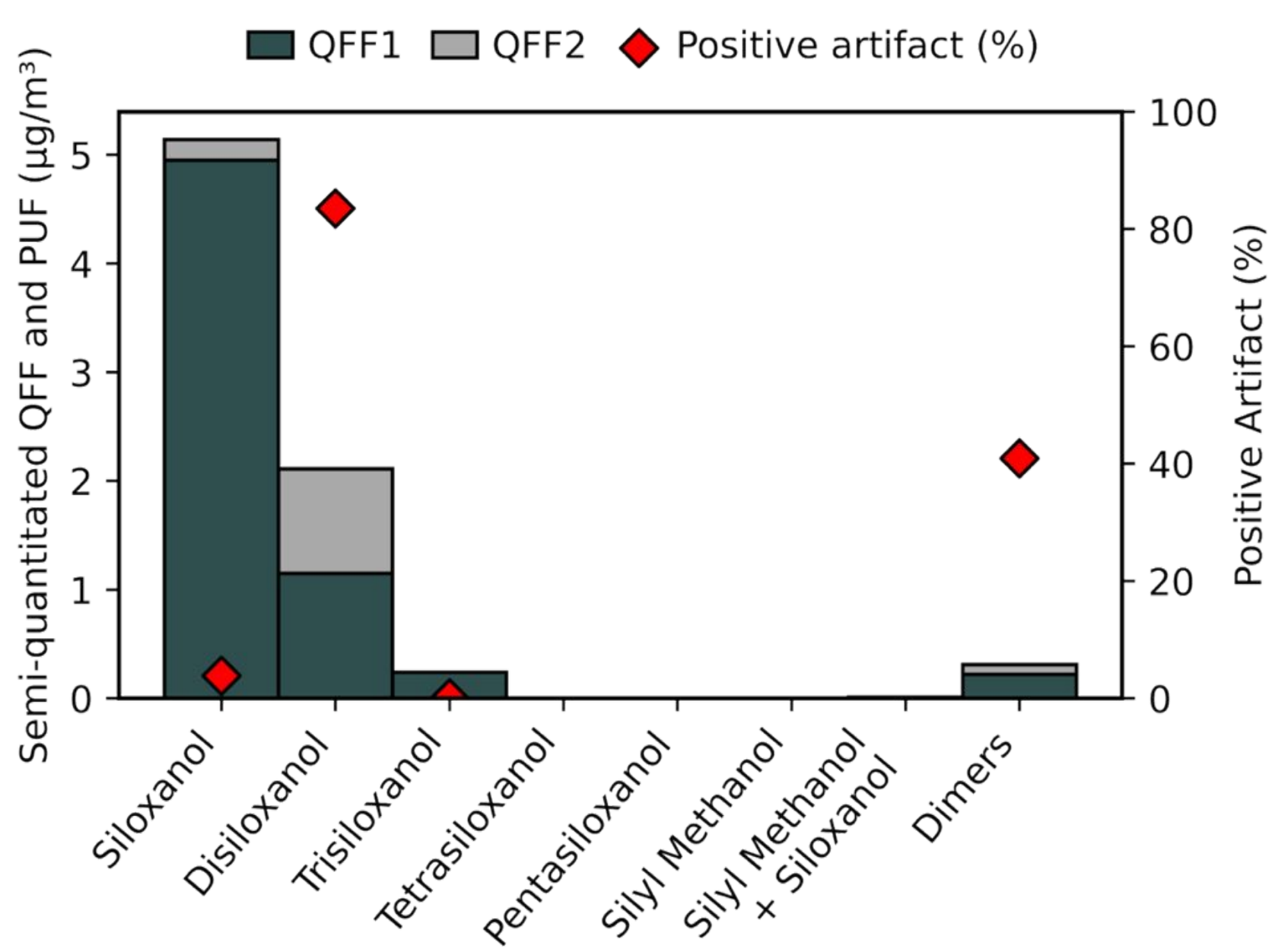

Figure S10: Semi-quantified concentrations of D5 oxidation products collected on front (QFF1) and backup (QFF2) quartz fiber filters, and corresponding positive sampling artifacts for OFR Sample 17. Bars represent particulate- and gas phase concentrations (µg m^{-3}), while red diamonds indicate the percentage of positive

artifact. The results show that siloxanol and disiloxanol dominate the particle-associated mass, with positive artifacts generally compound-specific, highest for disiloxanol and dimer species.

Because siloxanols can adsorb to QFFs and produce positive sampling artifacts, "particle phase" masses represent condensed phase SOA plus some adsorbed gases. Because only one tandem-filter (QFF–QFF) sample was collected in this study, and it exhibited compound-dependent artifacts, these observations cannot be generalized across the full set of OFR experiments, and no artifact correction was applied beyond that single sample.

S9. Kinetic Box Model

Simulation parameters are listed in Table S6-S8.

Table S6. non-VMS (1-67) reactions used in the kinetic box model mechanism

Reaction #	Reactant1	Reactant2	Product1	Product2	Product3	k
**1**	H2O2	HV185	HO2	H		1.00E-19
**2**	H2O2	HV254	OH	OH		6.70E-20
**3**	HNO2	HV185	OH	NO		9.00E-19
**4**	HNO2	HV254	OH	NO		1.45E-19
**5**	O3	HV254	O1D	O2		1.03E-17
**6**	NO2	HV185	NO	O1D		6.88E-18
**7**	NO2	HV254	NO	O3P		1.05E-20
**8**	HO2	HV185	OH	O1D		3.68E-18
**9**	HO2	HV254	OH	O1D		2.63E-19
**10**	O1D	H2O	OH	OH		1.99E-10
**11**	O1D	N2	O3P	N2		3.10E-11
**12**	OH	H2O2	H2O	HO2		1.80E-12
**13**	OH	NO	HNO2			7.20E-12
**14**	OH	HNO3	HO2	NO3		1.49E-13
**15**	HO2	NO2	HNO4			1.10E-12
**16**	NO2	NO3	N2O5			1.16E-12
**17**	N2O5		NO2	NO3		8.10E-02
**18**	O3P	O2	O3			1.43E-14
**19**	O3P	NO	NO2			1.61E-12
**20**	OH	HO2	H2O	O2		1.10E-10
**21**	HO2	HO2	H2O2	O2		3.60E-12
**22**	OH	O3	HO2	O2		7.50E-14
**23**	HO2	O3	OH	O2	O2	1.96E-15
**24**	O1D	O2	O3P	O2		3.96E-11
**25**	O1D	O3	O2	O2		1.20E-10

**26**	O1D	O3	O2	O3P	O3P	1.20E-10
**27**	O3P	OH	O2	H		2.68E-11
**28**	O3P	HO2	OH	O2		5.83E-11
**29**	O3P	H2O2	OH	HO2		1.83E-15
**30**	O3P	O3	O2	O2		8.56E-15
**31**	H	O3	OH	O2		2.94E-11
**32**	HO2	NO	OH	NO2		8.09E-12
**33**	H	HO2	OH	OH		7.20E-11
**34**	H	HO2	O3P	H2O		1.60E-12
**35**	H	HO2	O2	H2		6.90E-12
**36**	OH	H2	H2O	H		7.10E-15
**37**	OH	OH	H2O	O3P		1.80E-12
**38**	O3P	NO2	NO	O2		1.02E-11
**39**	O3P	NO2	NO3			3.19E-12
**40**	H	NO2	OH	NO		1.35E-10
**41**	OH	NO3	HO2	NO2		2.00E-11
**42**	HO2	NO3	OH	NO2	O2	3.50E-12
**43**	NO	NO3	NO2	NO2		2.57E-11
**44**	NO3	NO3	NO2	NO2	O2	2.49E-16
**45**	HNO4		NO2	HO2		9.00E-02
**46**	N2O5	H2O	HNO3	HNO3		1.00E-21
**47**	NO	O3	NO2	O2		2.06E-14
**48**	NO2	O3	NO3	O2		3.52E-17
**49**	OH	NO2	HNO3			1.05E-11
**50**	OH	HNO4	H2O	O2	NO2	4.59E-12
**51**	H	O2	HO2			9.25E-13
**52**	CH2O	OH	HO2	CO	H2O	9.19E-12
**53**	CO	OH	CO2	HO2		2.08E-13
**54**	O1D	CO2	CO2	O3P		1.10E-10
**55**	O1D	H2	OH	H		1.20E-10
**56**	O1D	N2	N2O			6.71E-17
**57**	OH	OH	H2O2			6.18E-12
**58**	O2	HV185	O3P	O3P		1.10E-20
**59**	H2O	HV185	OH	H		6.78E-20
**60**	O3P	NO3	O2	NO2		1.00E-11
**61**	OH	HNO2	H2O	NO2		4.93E-12
**62**	NO2	NO3	NO	NO2	O2	6.86E-16
**63**	O3	HNO2	O2	HNO3		2.50E-19

**64**	NO3	HVVIS	NO2		5.80E-19
**65**	N2O	HV185	N2	O1D	1.43E-19
**66**	N2O	O1D	N2	O2	4.96E-11
**67**	N2O	O1D	NO	NO	7.76E-11

Table S7: D5 oxidation, siloxanol formation, and multi-generational aging reactions implemented in the kinetic model. Reactions produce surrogate semi-volatile species (SV1–SV6) and simple siloxanols (SILOX1–SILOX5), which undergo subsequent OH-initiated aging. Branching and stoichiometric coefficients for these reactions are defined in the Section 2.4 (equations R1–R5).

Reactant1	Reactant2	Product 1	Product 2	Product 3	Product4-7	Product 8	Rate Coef.
D5	OH	VMSRO2	α_1(1-δ) SV1	α_2SV2	α_iSV3-6	α_1δ SILOX1	2.00E-12
SILOX1	OH	VMSRO2	β VVMSP‡	b SILOX2	c SV2		2.17E-12
SILOX2	OH	VMSRO2	β VVMSP	b SILOX3	c SV3		2.17E-12
SILOX3	OH	VMSRO2	β VVMSP	b SILOX4	c SV4		2.17E-12
SILOX4	OH	VMSRO2	β VVMSP	b SILOX5	c SV5		2.17E-12
SILOX5	OH	VMSRO2	β VVMSP	(1-β) SV6			2.17E-12
SV1	OH	VMSRO2	β VVMSP	(1-β) SV2			2.17E-12
SV2	OH	VMSRO2	β VVMSP	(1-β) SV3			2.17E-12
SV3	OH	VMSRO2	β VVMSP	(1-β) SV4			2.17E-12
SV4	OH	VMSRO2	β VVMSP	(1-β) SV5			2.17E-12
SV5	OH	VMSRO2	β VVMSP	(1-β) SV6			2.17E-12

‡ VVMSP denotes **V**olatile **VMS P**roduct, which is excluded from semivolatile partitioning.
δ is a siloxanol branching coefficient and β controls fragmentation to volatile products. b = δ (1-β); c = 1 - δ + δβ – β
α_1 = 0.89
α_2 = 2.913E-5
α_3 = 8.674E-2
α_4 = 1.373E-2
α_5 = 2.32E-3
α_6 = 1.237E-4

Table S8: D5 RO2 fate reactions used to diagnose RO2 loss pathways in the kinetic model described in Section 2.4 (equations R7-11). These reactions are tracked during model integration and do not directly affect product distributions or aerosol formation.

Reactant1	Reactant2	Product1	Product2	Rate Coef.
VMSRO2	NO	SINK	NO2	9.00E-12
VMSRO2	HO2	SINK		1.70E-11
VMSRO2	VMSRO2	SINK		1.00E-13
VMSRO2	OH	SINK		1.00E-10
VMSRO2		SINK		8.00E-03

The following statistical measures, (Eq. S4-6), were used to evaluate model-measurement agreement:

$$MB = \frac{1}{N} \sum_{i=1}^{N} (M_i - O_i) \qquad \text{(Eq. S4)}$$

$$ME = \frac{1}{N} \sum_{i=1}^{N} |M_i - O_i| \qquad \text{(Eq. S5)}$$

$$ME = \frac{1}{N} \sum_{i=1}^{N} \frac{M_i - O_i}{O_i} \qquad \text{(Eq. S6)}$$

Where MB is the mean bias between modeled and observed values.
ME is the mean error between modeled and observed values.
M_i is the modeled value for the i-th observation.
O_i is the observed (measured) value for the i-th observation.
N is the total number of paired modeled and observed data points.
$|M_i - O_i|$ represents the absolute error.
$\frac{M_i - O_i}{O_i}$ is the fractional error normalized by the observed value.

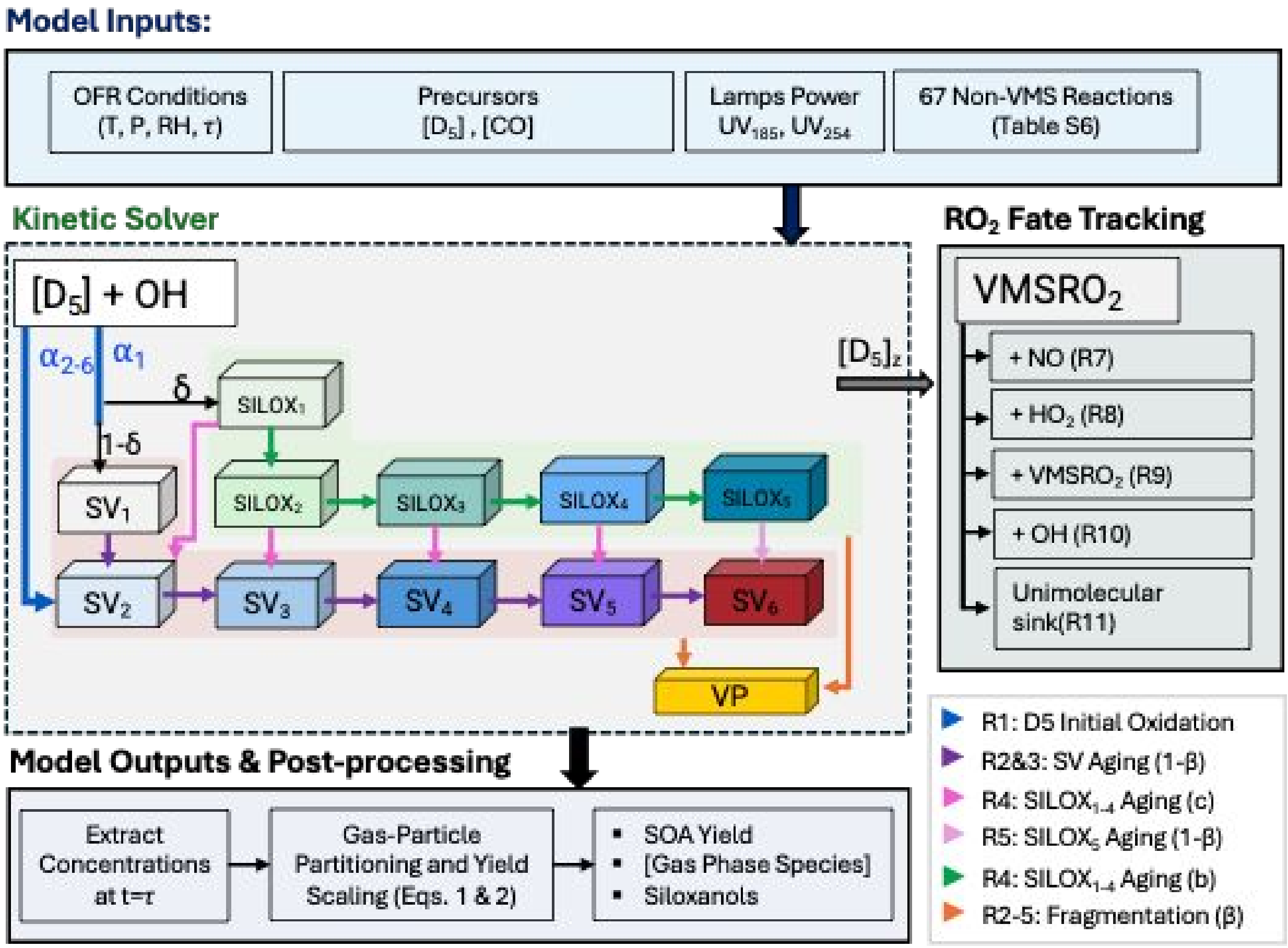

Figure S11: Schematic of the OFR kinetic box model. The framework integrates UV-driven radical chemistry with oxidation mechanism for explicit siloxanols ($SILOX_{1\text{-}5}$) and lumped VBS surrogates ($SV_{1\text{-}6}$). Branching and fragmentation parameters govern product distribution, while post-processing utilizes gas-particle partitioning and yield scaling to determine final aerosol mass.

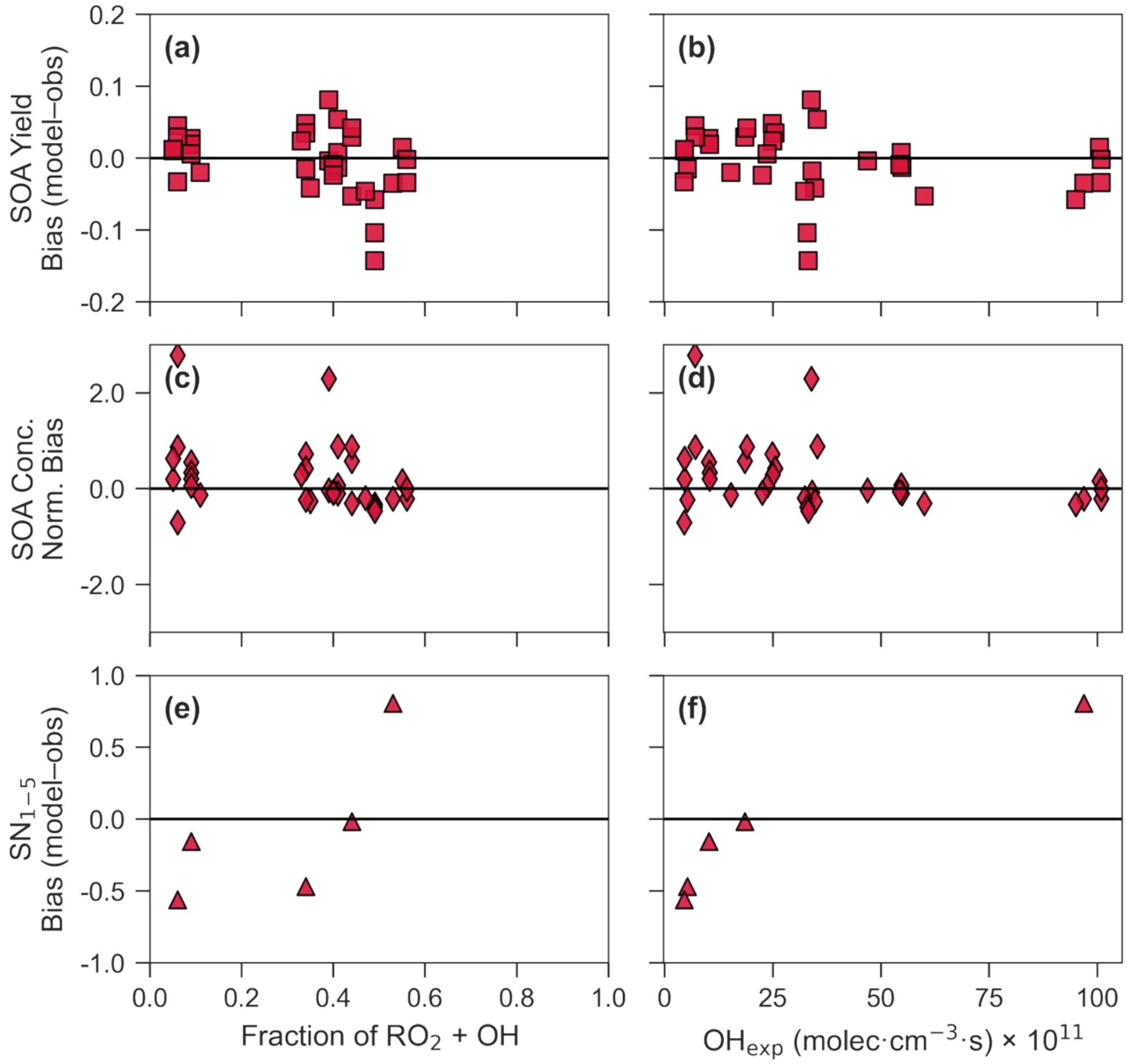

Figure S12: Panels (a,b) show bias in SOA yield prediction as a function of RO_2 fate and OH_{exp}, respectively. Panels (c,d) show normalized bias in outlet aerosol concentration as a function of RO_2 fate and OH_{exp}, with no significant trends in either case. Panels (e,f) show bias versus the model siloxanol number SN_{1-5}; no significant dependence on RO_2 fate is observed, while a statistically significant dependence on OH_{exp} is present.

Table S9: Results of linear regression of kinetic model residuals. Regression pValues of less than 0.05 are bold and italic, and significant variables (P<0.05) within significant models also bold and italic.

Model	Variable	Estimate	Standard Error	tStat	pValue
$Y1^a_{resid}$ (RO_2 fate)<br>34 obs, 32 DOF, RMSE: 0.0428, R^2: 0.121, R^2_a: 0.0936, F-stat: 4.41, ***p = 0.0438***					
	Intercept	0.0245	0.0157	1.56	0.128
	RO_2 fate	-0.0889	0.0424	-2.10	***0.044***
$Y2^a_{resid}$ (RO_2 fate)<br>34 obs, 32 DOF, RMSE: 0.693, R^2: 0.085, R^2_a: 0.0564, F-stat: 2.97, p = 0.0943					
	Intercept	0.626	0.254	2.47	0.0192
	RO_2 fate	-1.18	0.685	-1.72	0.0943
$Y3^a_{resid}$ (RO_2 fate)<br>5 obs, 3 DOF, RMSE: 0.433, R^2: 0.522, R^2_a: 0.363, F-stat: 3.28, p = 0.168					
	Intercept	-0.624	0.358	-1.74	0.180

	RO_2 fate	1.87	1.03	1.81	0.168
$Y1_{resid}$ (OH_{exp})<br>34 obs, 32 DOF, RMSE: 0.044, R^2: 0.075, R^2_a: 0.0461, F-stat: 2.6, p = 0.117					
	Intercept	0.363	0.228	1.59	0.122
	OH_{exp}	-0.0297	0.0184	-1.61	0.117
$Y2_{resid}$ (OH_{exp})<br>34 obs, 32 DOF, RMSE: 0.696, R^2: 0.0781, R^2_a: 0.0493, F-stat: 2.71, p = 0.109					
	Intercept	6.19	3.62	1.71	0.0966
	OH_{exp}	-0.480	0.291	-1.65	0.109
$Y3_{resid}$ (OH_{exp})<br>5 obs, 3 DOF, RMSE: 0.055, R^2: 0.992, R^2_a: 0.99, F-stat: 385, ***p = 2.89 × 10⁻⁴***					
	Intercept	-12.2	0.620	-19.7	***$2.85x10^{-4}$***
	OHexp	1.00	0.051	19.6	***$2.89x10^{-4}$***
$Y1_{resid}$ (RO_2 fate, OH_{exp})<br>34 obs, 31 DOF, RMSE: 0.0435, R^2: 0.123, R^2_a: 0.066, F-stat: 2.17, p = 0.132					
	Intercept	-0.0717	0.404	-0.177	0.860
	RO_2 fate	-0.105	0.0812	-1.30	0.204
	OH_{exp}	0.008	0.034	0.238	0.813
$Y2_{resid}$ (RO_2 fate, OH_{exp})<br>34 obs, 31 DOF, RMSE: 0.703, R^2: 0.0887, R^2_a: 0.0299, F-stat: 1.51, p = 0.237					
	Intercept	2.94	6.53	0.450	0.656
	RO_2 fate	-0.790	1.31	-0.600	0.553
	OH_{exp}	-0.197	0.556	-0.354	0.725
$Y3_{resid}$ (RO_2 fate, OH_{exp})<br>5 obs, 2 DOF, RMSE: 0.0607, R^2: 0.994, R^2_a: 0.987, F-stat: 158, ***p = 0.00628***					
	Intercept	-12.7	0.987	-12.9	***0.00598***
	RO_2 fate	-0.149	0.219	-0.680	0.566
	OH_{exp}	1.045	0.0853	12.3	***0.00659***

[a] Y1, Y2, and Y3, the regression outcome variables, are the bias in yield ($Y_{mod} – Y_{obs}$), normalized bias in aerosol concentration (C_{mod}-C_{obs})/C_{obs}, and bias in siloxanol number ($SN_{mod} – SN_{obs}$), respectively.

Interpretation of coefficients and slope:

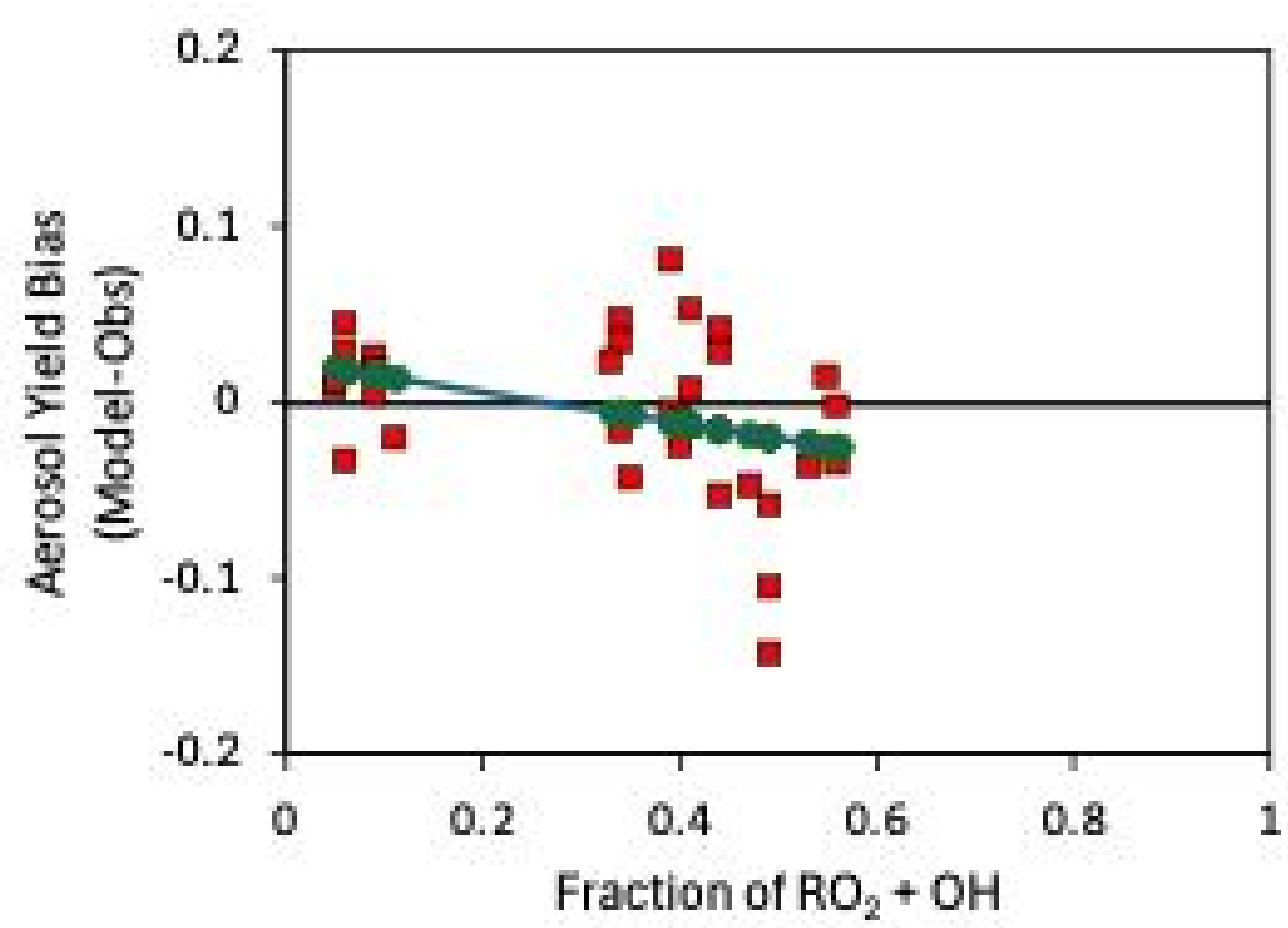

Figure S13: Linear regression result of residuals for case Y1resid (RO_2 fate). The slope coefficient is -0.09.

Under conditions dominated by $RO_2 + HO_2$ reactions ($RO_2 + OH < 0.2$, $RO_2 + HO_2 > 0.8$), the regression residuals are positive ($Y_{mod} – Y_{obs} > 0$), indicating systematic model overprediction of aerosol yield. In

contrast, under conditions where RO_2 + OH and RO_2 + HO_2 pathways contribute comparably (RO_2 + OH = 0.4 – 0.6, RO_2 + HO_2 = 0.4 – 0.6), the residuals are negative ($Y_{mod} - Y_{obs} < 0$), indication Model underprediction.

These trends suggest that RO_2 + OH pathway may lead to enhanced aerosol yield relative to RO_2 + HO_2-dominated conditions. However, this regression result becomes statistically insignificant when both OH_{exp} and RO_2 fate are included simultaneously as predictors in the regression model. Although not shown, the sign of the slope associated with the RO_2 fate remains consistent when the analysis is restricted to lower OH_{exp} cases and higher OH_{exp} cases. The results in the table and figure are for all OH_{exp}.

S10. OFR 185 nm and 254 nm Lamps Power and Photon Flux

The kinetic box model incorporated photon flux values from low-pressure mercury UV lamps installed inside the OFR, which emit simultaneously at 254 nm and 185 nm wavelengths. During experiments, humidified air containing water vapor was passed through the OFR operated in the OFR185 mode. In this configuration, ozone was photochemically generated within the reactor via HO_2 photolysis initiated by 185 nm photons.

Table S10. Lamp voltage (0–10 V), irradiance (µW/cm²), and normalized power output used in kinetic model input.

Lamps Voltage	Irradiance (µW cm^{-2})	Normalized Lamp power
**10**	1,520	1.00
**8**	1,500	0.99
**7**	1,400	0.92
**6**	1,300	0.86
**5**	1,100	0.72
**4**	900	0.59
**3**	580	0.38
**2**	200	0.13
**1**	5	0.00
**0**	0	0.00

The OFR setup utilized UV lamps (model GPH436T5/VH/4P 254nm/185nm Splice UV Lamp, HSM-UNV-214-LT5 ballast) with a dimming voltage range of 1–10 VDC, providing normalized UV output monitored by a UV sensor. Lamp voltages were adjusted to control irradiance, achieving a normalized power output range of 0 to 1 (0–100%). To estimate lamp power output across different dimming voltage ranges, two polynomial equations were derived due to the nonlinearity of the dimming-voltage-to-output relationship at higher voltages. These equations, shown in Figure S15, provided precise control of lamp power to achieve desired experimental condition:

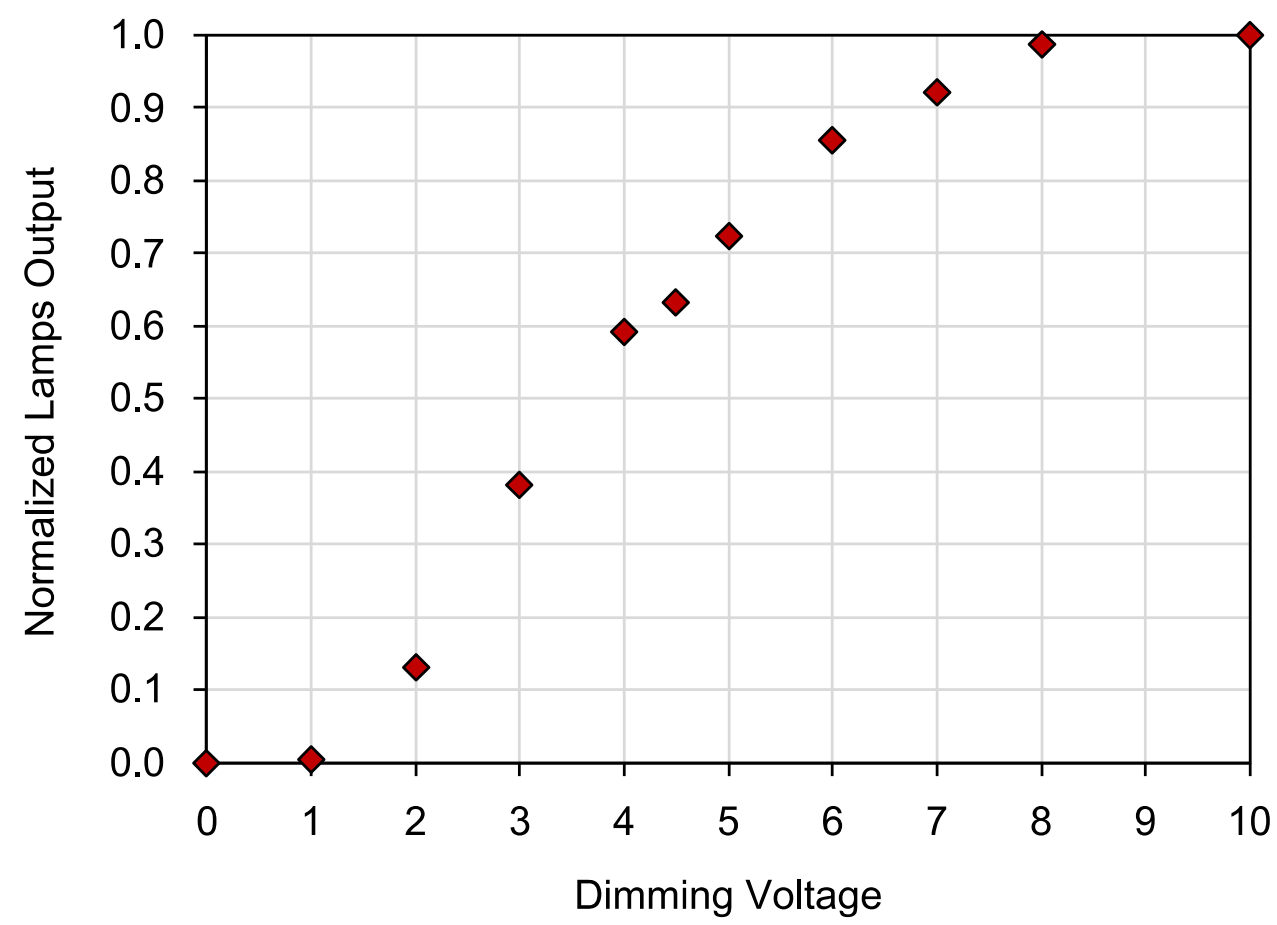

Figure S14. The normalized UV output level (1-100%) as a function of dimming voltage (1-10 VDC) applied to the lamps in the OFR.

(a) (b)

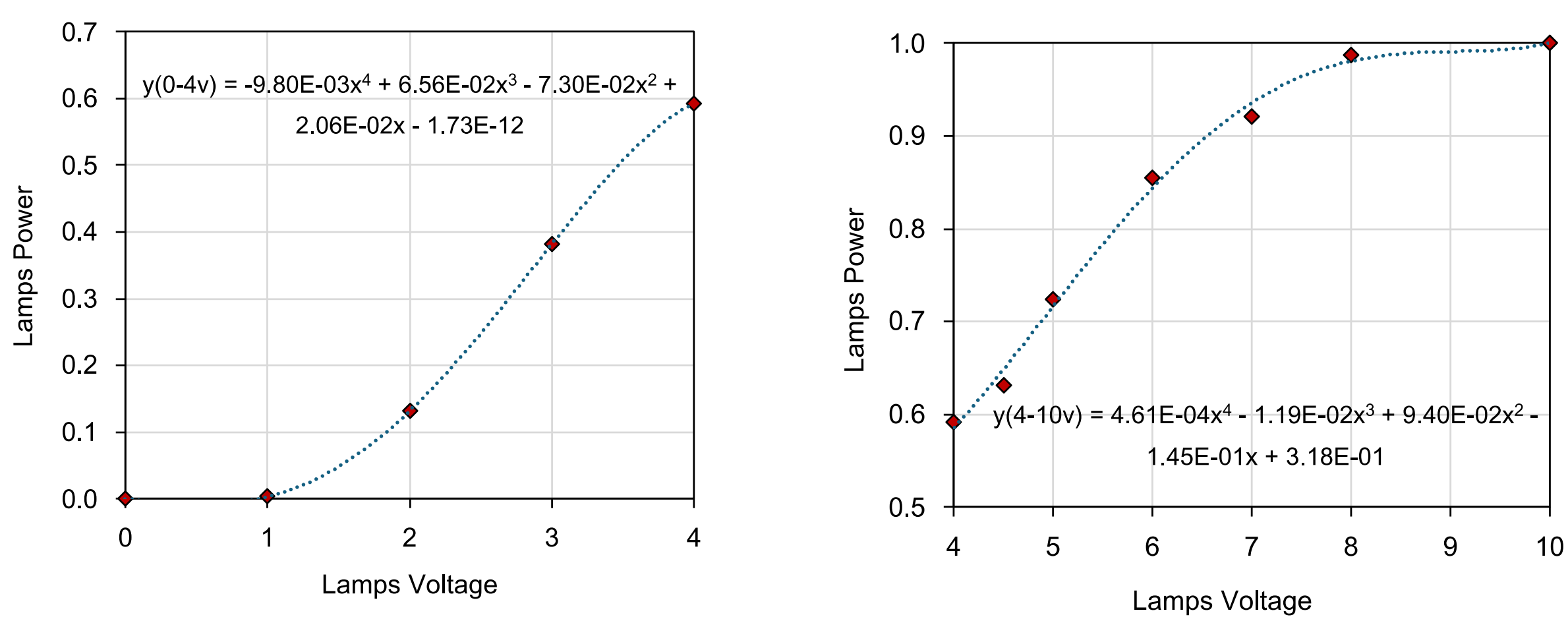

Figure S15. Polynomial relationships between lamp voltage and normalized lamp power output for the two voltage ranges: 0–4 V (panel a) and 4–10 V (panel b). These curves illustrate the nonlinear behavior of lamp power with respect to voltage, with polynomial equations derived to accurately estimate normalized power for Chem-Kin model calibration and photon flux calculations.

As presented in Table S10, UV irradiance measurements under varying lamp power settings were converted to photon flux values for 254 nm by dividing the measured irradiance by the photon energy at λ = 254 nm. The calculated photon fluxes were compared with the maximum UV254 flux reported by Lambe et al. (2019) and Rowe et al. (2020), 3.5×10^{15} photons cm^2 s for two lamps and 7.0×10^{15} photons cm^2 s four lamps[4, 5], to ensure consistency with established OFR operating conditions. UV185 flux was estimated using an initial UV185/UV254 ratio range of 0.04 to 0.1 and the O_3 concentration as model output variable was compared with O_3 levels at the downstream of the OFR with identical experimental condition. The best alignment between modeled and measured O_3 values were achieved the maximum photon fluxes of 7.0×10^{15} photons cm^2 s for UV254 and 3.2×10^{14} photons cm^2 s for UV185, corresponding to the UV185/UV254 ratio of 0.045.

S11. New York City OH_{exp} Estimation

To compare oxidation conditions in the OFR samples with the ambient samples collected between July 25 and July 29, 2022 in New York City (see Table S4 for sampling start and end times), ambient hourly OH_{exp} was estimated using the toluene:benzene chemical clock method, following Warneke et al. (2007), de Gouw et al. (2017), and Doak et al. (2021).[6-8] Benzene and toluene concentrations were from EPA Air Quality System (AQS) measurements (https://aqs.epa.gov/aqsweb/airdata). Hourly benzene and toluene concentrations in New York City during the July 25–28, 2022 sampling period were retrieved from the 2022 AQS VOC archive. The rate coefficients, governing equation, and procedure for determining the fresh-emission ratio are described in Doak et al. (2021).[9]

It is important to note that OH_{exp} is not a single, well-defined quantity, but rather a distribution describing the range of OH histories experienced by individual molecules, here D5. Because the true OH_{exp} distribution for D5 cannot be measured directly in ambient air, we use the mean value inferred from a benzene:toluene chemical clock as a proxy, recognizing that this introduces uncertainty because benzene and toluene emissions do not necessarily follow the same temporal or spatial patterns as D5. However, this method remains the most widely applied and observationally constrained approach for estimating ambient oxidation conditions.

Across the sampling periods, ambient OH_{exp} estimated approximately 6.4×10^{10} to 1.6×10^{11} molecule cm^{-3} s (25th–95th percentile), with a mean value of 9.0×10^{10} molecule cm^{-3} s. Acknowledging the uncertainties discussed above, we used the mean OH_{exp} as a representative ambient oxidation level for graphs comparing composition metrics as a faction of OH_{exp}.

References

(1) Brunet, C. E.; Marek, R. F.; Stanier, C. O.; Hornbuckle, K. C. Concentrations of Volatile Methyl Siloxanes in New York City Reflect Emissions from Personal Care and Industrial Use. *Environmental Science & Technology* **2024**, *58* (20), 8835-8845. DOI: 10.1021/acs.est.3c10752.

(2) Welker, J. K.; Meepage, J. N.; Stanier, C. O.; Stone, E. A. Gas-Particle Distribution of D5 Oxidation Products in New York City during Summertime. *Environmental Science & Technology* **2025**, *2* (11), 2517-2526. DOI: 10.1021/acsestair.5c00193.

(3) Meepage, J. N.; Welker, J. K.; Meyer, C. M.; Mohammadi, S.; Stanier, C. O.; Stone, E. A. Advances in the Separation and Detection of Secondary Organic Aerosol Produced by Decamethylcyclopentasiloxane (D5) in Laboratory-Generated and Ambient Aerosol. *Environmental Science & Technology* **2024**, *1* (5), 365-375. DOI: 10.1021/acsestair.3c00073.

(4) Lambe, A. T.; Krechmer, J. E.; Peng, Z.; Casar, J. R.; Carrasquillo, A. J.; Raff, J. D.; Jimenez, J. L.; Worsnop, D. R. HOx and NOx production in oxidation flow reactors via photolysis of isopropyl nitrite, isopropyl nitrite-d7, and 1,3-propyl dinitrite at λ = 254, 350, and 369 nm. *Atmospheric Measurement Techniques* **2019**, *12* (1), 299-311. DOI: 10.5194/amt-12-299-2019.

(5) Rowe, J. P.; Lambe, A. T.; Brune, W. H. Technical Note: Effect of varying the λ = 185 and 254 nm photon flux ratio on radical generation in oxidation flow reactors. *Atmospheric Chemistry and Physics* **2020**, *20* (21), 13417-13424. DOI: 10.5194/acp-20-13417-2020.

(6) de Gouw, J. A.; Gilman, J. B.; Kim, S.-W.; Lerner, B. M.; Isaacman-VanWertz, G.; McDonald, B. C.; Warneke, C.; Kuster, W. C.; Lefer, B. L.; Griffith, S. M.; et al. Chemistry of Volatile Organic Compounds in the Los Angeles basin: Nighttime Removal of Alkenes and Determination of Emission Ratios. *Journal of Geophysical Research: Atmospheres* **2017**, *122* (21). DOI: 10.1002/2017JD027459.

(7) Doak, A. G.; Christiansen, M. B.; Alwe, H. D.; Bertram, T. H.; Carmichael, G.; Cleary, P.; Czarnetzki, A. C.; Dickens, A. F.; Janssen, M.; Kenski, D.; et al. Characterization of ground-based atmospheric pollution and meteorology sampling stations during the Lake Michigan Ozone Study 2017. *Journal of the Air & Waste Management Association* **2021**, *71* (7), 866-889. DOI: 10.1080/10962247.2021.1900000.

(8) Warneke, C.; McKeen, S. A.; de Gouw, J. A.; Goldan, P. D.; Kuster, W. C.; Holloway, J. S.; Williams, E. J.; Lerner, B. M.; Parrish, D. D.; Trainer, M.; et al. Determination of urban volatile organic compound emission ratios and comparison with an emissions database. *Journal of Geophysical Research: Atmospheres* **2007**, *112* (D10). DOI: 10.1029/2006JD007930.

(9) Doak, A. G.; Christiansen, M. B.; Alwe, H. D.; Bertram, T. H.; Carmichael, G.; Cleary, P.; Czarnetzki, A. C.; Dickens, A. F.; Janssen, M.; Kenski, D. Characterization of ground-based atmospheric pollution and meteorology sampling stations during the Lake Michigan Ozone Study 2017. *Journal of the Air & Waste Management Association* **2021**, *71* (7), 866-889.
